# Bone adhesive with temporally-synchronized degradation for enhanced osteointegration

**DOI:** 10.1038/s41413-026-00522-8

**Published:** 2026-04-07

**Authors:** Jun-ting Gu, Zhi-ting Li, Yu-zhu Wang, Dong-xiao Hao, Gao-peng Dang, Xiao-Qing Cao, Franklin R. Tay, Ji-hua Chen, Conrado Aparicio, Kai Jiao, Li-na Niu

**Affiliations:** 1 National Clinical Research Center for Oral Diseases & State Key Laboratory of Military Stomatology & Shaanxi Key Laboratory of Stomatology, School of Stomatology, The Fourth Military Medical University, Xi’an, Shaanxi China; 2 National Translational Science Center for Molecular Medicine, Department of Cell Biology, State Key Laboratory of Cancer Biology, The Fourth Military Medical University, Xi’an, Shaanxi China; 3 The Third Affiliated Hospital of Xinxiang Medical University, Xinxiang, Henan China; 4 Dental College of Georgia, Augusta University, Augusta, GA USA; 5 Catalan Institute for Research and Advanced Studies (ICREA), Barcelona, Spain; 6 BOBI- Bioinspired Oral Biomaterials and Interfaces, Department of Materials Science and Engineering, EEBE, Technical University of Catalonia (UPC)-Barcelona Tech, Barcelona, Spain; 7 Department of Stomatology, Tangdu Hospital, The Fourth Military Medical University, Xi’an, Shaanxi China

**Keywords:** Bone, Bone quality and biomechanics

## Abstract

Bone adhesives have emerged as promising alternatives for complex fracture fixation. However, discrepancies between material degradation rates and the physiological timeline of bone healing remain a critical limitation. Here, a polyurethane-based adhesive (TNC) was developed, synthesized from trimeric hexamethylene diisocyanate, nano-hydroxyapatite, and type I collagen. The TNC demonstrates strong initial adhesion to both wet and blood-contaminated bone surfaces and exhibits excellent biocompatibility. A distinguishing feature of TNC is its capacity to synchronize degradation with the stages of bone healing. During degradation, TNC forms a mineralized surface layer that releases calcium ions. The calcium ions activate cathepsin K, an enzyme integral to bone remodeling. This calcium-mediated mechanism accelerates TNC degradation by 1.9-fold during the remodeling phase compared to the initial phase. In a rat skull fracture model, TNC supported effective fracture stabilization and achieved favorable bone regeneration at 8 weeks after implantation. These findings demonstrate that TNC combines early mechanical stability with phase-specific degradability to facilitate bone regeneration in a temporally-controlled manner. The present work provides a framework for the development of bio-responsive bone adhesives that synchronize degradation behavior with healing phases for orthopedic applications.

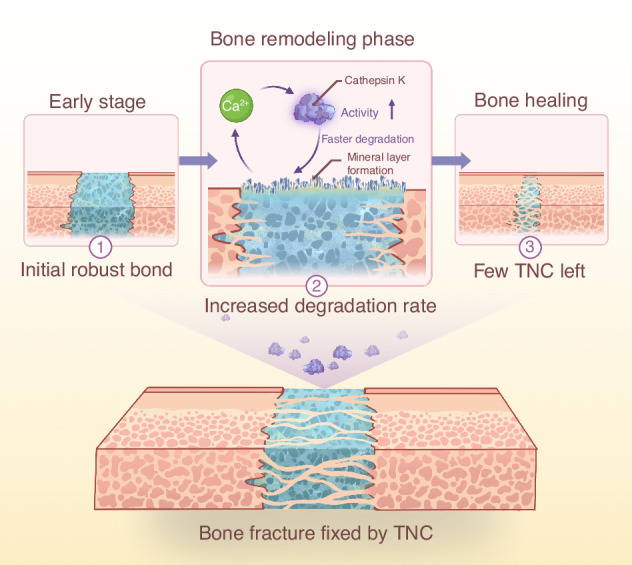

## Introduction

Bone adhesive refers to a material that is capable of stabilizing bone fractures through adhesion while simultaneously supporting the healing process. The concept was inspired by general tissue adhesives and was first proposed by Gluck et al. in 1890.^1^ Compared to internal metal fixation, bone adhesives offer improved convenience and reduce localized stress concentrations. This field has gained increasing attention recently in orthopedic biomaterials research.

The polyurethane-based adhesive Kryptonite was the first bone adhesive approved by the United States Food and Drug Administration. Kryptonite showed potential for sternal fracture fixation because of its high bonding strength.^2^ However, it was recalled in 2012 due to prolonged setting time and suboptimal degradation behavior.^2,3^ Other materials labeled as bone adhesives include poly(methyl methacrylate) and calcium phosphate bone cements. These materials exhibit limited interfacial adhesion to bone and hence do not qualify as true bone adhesives.^4^ Commercially available tissue adhesives, such as fibrin glue and cyanoacrylate adhesives, provide adequate soft tissue bonding but lack sufficient strength for fixation of bone fragments.^5,6^ Currently, there is no clinically available bone adhesive that fully satisfies the requirements for effective bone stabilization.

An ideal bone adhesive should demonstrate robust and durable adhesion, along with high biocompatibility and bioactivity to support tissue integration and minimize cytotoxicity.^4,7–9^ In addition, controlled degradability is essential because premature degradation may result in fragment instability. In contrast, delayed degradation can obstruct callus formation.^10^ Therefore, precise temporal coordination of adhesive degradation with bone healing remains a critical challenge.

Bone healing proceeds through distinct biological phases: hematoma formation, fibrous callus development, bony callus formation, and subsequent remodeling.^11,12^ Effective adhesion is required during the early phases to ensure fragment stability. Once bony bridging is established, degradation of the adhesive is preferred to enable remodeling. Ideally, the degradation rate should be slow during early healing and accelerate during the remodeling phase.

During remodeling, cathepsin K is secreted by osteoclasts to facilitate the resorption of collagen type I and nano-hydroxyapatite, the primary components of the callus matrix.^13,14^ This enzyme is also involved in the degradation of polymers such as polyurethane. Adhesives containing nano-hydroxyapatite can generate a mineral layer during degradation to enhance calcium release.^10,15^ Owing to the calcium-binding sites in cathepsin K,^16^ this release may potentiate enzymatic activity and thereby accelerate degradation during bone remodeling.

In response to this biological context, a polyurethane-based adhesive (TNC) was developed, consisting of trimeric hexamethylene diisocyanate (tri-HDI), nano-hydroxyapatite, and collagen type I. It is hypothesized that TNC provides immediate mechanical stabilization and subsequently undergoes calcium-mediated, cathepsin K-responsive degradation in vivo to support temporally-controlled resorption and improved bone regeneration. This hypothesis was evaluated through a combination of in vitro degradation studies, molecular dynamics simulations, and in vivo cranial fracture repair in a rat model.

## Results

### Synthesis of TNC

**Fig. 1:**
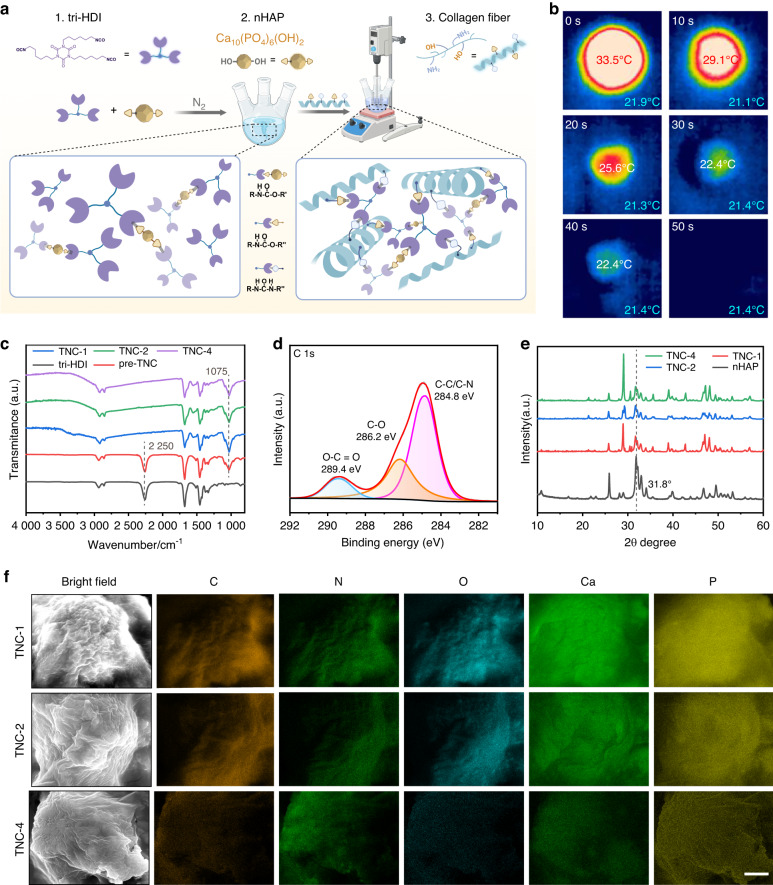
Preparation of TNC. **a** Schematic of of the synthesis process. **b** Thermal images captured during in-situ polymerization of TNC-1 on a silicone mold at room temperature. **c** Infrared spectra comparing tri-HDI, pre-TNC, and TNC samples with varying collagen content. **d** XPS full spectrum and high-resolution C 1s spectrum of TNC. **e** XRD patterns of nano-hydroxyapatite powder and TNC. **f** EDX mapping showing the distribution of C, N, O, Ca, and P on the TNC surface. Scale bar: 5 μm

The composite material TNC was a polyurethane-based adhesive composed of collagen type I and nano-hydroxyapatite, which are linked through tri-HDI. Specimens of TNC containing 1%, 2%, and 4% collagen by mass were designated as TNC-1, TNC-2, and TNC-4, respectively. In the initial synthesis step, tri-HDI reacted with nano-hydroxyapatite to form a prepolymer (pre-TNC). Catalyzed by dibutyltin dilaurate, the -NCO groups of tri-HDI reacted with the -OH groups of nano-hydroxyapatite to form urethane bonds (-NHCOO-). This interaction was confirmed by the appearance of a peak at ~1 075 cm⁻¹ (-C-O-) in the infrared spectrum of pre-TNC, as determined by fourier transform infrared spectroscopy (FTIR). Residual -NCO groups (~2 250 cm⁻¹) in the pre-TNC (Fig. 1c) enabled subsequent reactions with the -OH and -NH₂ groups in collagen to produce additional urethane and urea linkages (Fig. 1a, c).

The reaction between -NCO and -NH₂ occurs rapidly without the need for a catalyst. This characteristic enables TNC to polymerize at room temperature. Because the peak polymerization temperature of TNC was approximately 35 °C (Fig. 1b, Fig. S1c, supplementary information), this minimized the risk of thermal tissue damage commonly associated with bone cements.^17^ During curing, TNC transitioned from a viscous liquid to a porous white solid, reaching full polymerization within 10 min (Fig. S1a, S1b). The timeframe was consistent with clinical applicability.^10^ Examination with FTIR confirmed the complete consumption of -NCO groups (Fig. 1c), thereby reducing the risk of residual toxicity during the application of TNC in vivo.^18^ X-ray photoelectron spectroscopy (XPS) further verified successful polymerization (Figs. 1d and S2). X-ray diffraction (XRD) analysis revealed the retention of the crystalline peak of nano-hydroxyapatite at 2θ = 31.8° (Fig. 1e). Scanning electron microscopy-energy dispersive X-ray analysis demonstrated uniform distribution of calcium and phosphorus ions throughout the composite (Fig. 1f).

### Characterization

**Fig. 2:**
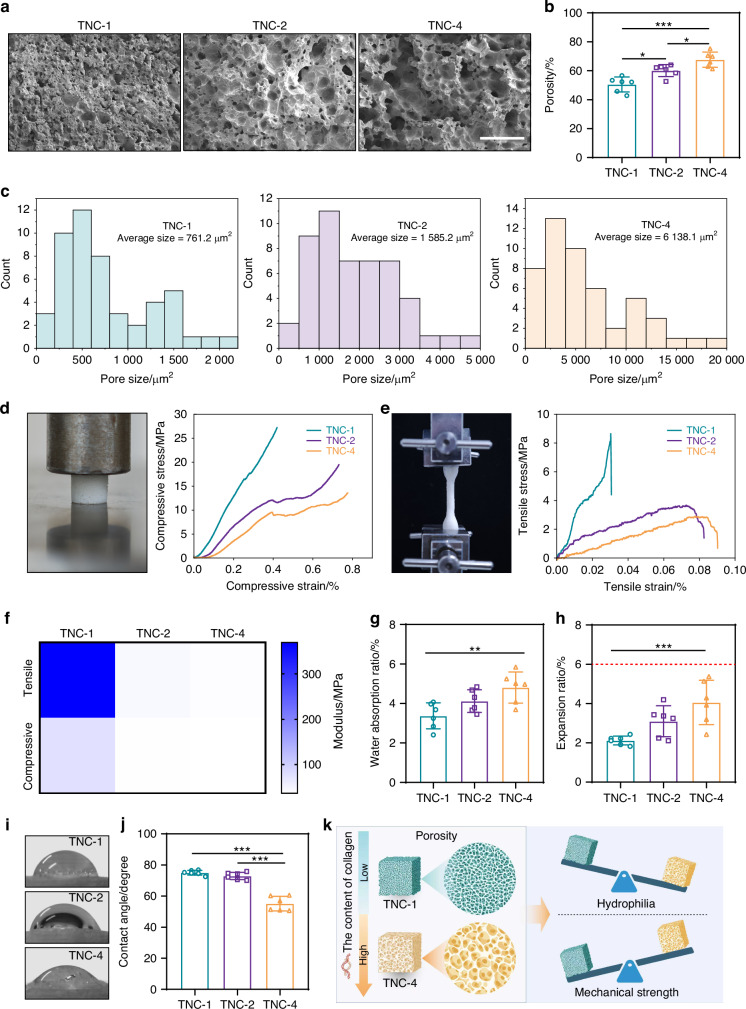
Characteristic of TNC. **a** SEM image, (**b**) porosity (*n* = 6) and (**c**) pore size of TNC with different collagen proportions. Scale bar: 500 μm. Photograph and representative curve of (**d**) compressive and (**e**) tensile test. **f** The compressive and tensile modulus of TNC (*n* = 6). **g** Water absorption ratio and (**h**) expansion ration of TNC (*n* = 6), the red dotted line represents the 6% expansion ratio. **i** Representative image and (**j**) contact angle of TNC (*n* = 6). **k** Summary of the changing trend of TNC physical properties with the variation of collagen components. *P < 0.05; **P < 0.01; ***P < 0.001

The TNC absorbed ambient moisture during curing. The moisture acted as a foaming agent for the polyurethane matrix, resulting in a porous structure (Figs. 2a, S3).^19^ This porosity facilitates cell infiltration and tissue regeneration. Both pore size and porosity increased with collagen content. The average pore size was 761.2 μm² in TNC-1, 1 585.2 μm² in TNC-2, and 6 138.1 μm² in TNC-4 (Fig. 2c); the corresponding porosity values were 50.5% ± 5.2%, 60.1% ± 4.1%, and 67.7% ± 5.2%, respectively (Fig. 2b). All pore sizes were sufficient to accommodate osteoblast infiltration (20–50 μm). The porosity values aligned with the 50%–90% range typical of cancellous bone.^20^

Mechanical strength is essential for the performance of bone adhesives. Among the formulations, TNC-1 exhibited the highest compressive and tensile moduli (Fig. 2d–f). Both properties declined with increasing collagen content. These properties may be attributed to increased porosity and reduced nano-hydroxyapatite loading, as the latter provides mechanical reinforcement.^21^ Water absorption remained low, and dimensional stability was maintained without significant swelling (Fig. 2g, h). These properties render TNC suitable for application in moist environments.^22,23^

Collagen content also affected surface hydrophilicity. The TNC-4 version demonstrated the lowest water contact angle (55.13° ± 4.68°). The low water contact angle is consistent with the hydrophilic nature of collagen and favors cell attachment and fluid transport (Fig. 2i, j).^24,25^ Thermogravimetric analysis revealed minimal differences in thermal decomposition temperatures among all groups, indicating comparable thermal stability (Fig. S4). Overall, TNC-4 showed the greatest promise for tissue healing due to enhanced porosity and hydrophilicity, while TNC-1 demonstrated superior mechanical performance (Fig. 2k). Consequently, all three formulations were selected for subsequent investigation.

### Adhesive performance

**Fig. 3:**
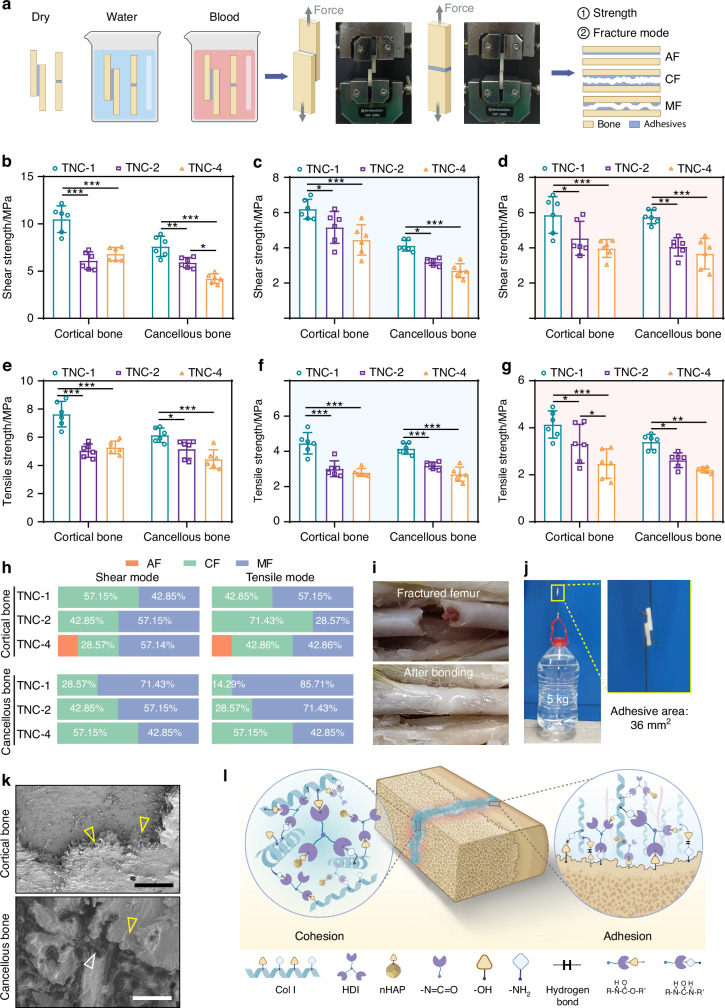
Adhesive behavior of TNC. **a** Schematics of the adhesive strength test. Shear strength of TNC in the dry (**b**), wet (**c**) and blood contaminated (**d**) cortical bone or cancellous bone interface (*n* = 6). Tensile strength of TNC in the dry (**e**), wet (**f**) and blood contaminated (**g**) cortical bone or cancellous bone interface (*n* = 6). **h** The fracture model of TNC. **i** Ex vivo rat femur fractures fixed by TNC-1. **j** Lift-up of 5 kg bucket using the TNC-1 glued cortical bone with 36 mm^2^ bonding area. **k** Representative SEM image of bonding interface. The yellow triangle represents the adhesive sample, and the white triangle represents cancellous bone. Scale bar: 250 μm. **l** Illustration depicting the cohesive of inner structure and adhesive interacting with bone tissue. *P < 0.05; **P < 0.01; ***P < 0.001

An important property of bone adhesives is their ability to generate robust adhesion to bone fragments.^26^ The adhesive performance of the developed material was evaluated on cancellous and cortical bone under ambient conditions (Fig. 3a). Among the three groups tested, TNC-1 exhibited the highest bond strength. On cortical bone, shear and tensile strengths reached (10.50 ± 1.41) MPa and (7.64 ± 0.90) MPa, respectively (Fig. 3b, e). On cancellous bone, TNC-1 achieved shear and tensile strengths of (7.61 ± 1.08) MPa and (6.15 ± 0.51) MPa, respectively (Fig. 3b, e). These values are consistent with, or superior to results previously reported (Fig. S5). For practical demonstration, rat femur bone segments were successfully reattached using TNC (Fig. 3i). In addition, bovine cortical bone specimens with a 36 mm² bonding area were able to support a ~5 kg load without detachment (Fig. 3j and Video S1). These results validated the mechanical stability of the bone adhesive.

Because bonding surfaces may be contaminated by water or blood during clinical use,^27^ the adhesive strength of TNC was also evaluated under such conditions (Fig. S6). As shown in Fig. 3b–g, all groups exhibited reduced performance in the presence of contaminants. Nevertheless, adhesive strength remained above 0.2 MPa, a threshold considered adequate for practical use.^8,28^

Fracture mode analysis provides additional insight into adhesive performance and reliability.^29^ Three failure modes were identified: adhesive failure (AF), cohesive failure (CF), and mixed failure (MF).^30^ In lap-shear and butt-joint testing, the mode proportions follow the expected mechanics of interface versus bulk. When adhesion is the limiting element, cracks initiate and propagate along the interface, yielding a higher AF fraction and a lower measured strength.^31^ This pattern is most evident in TNC-4 (Fig. 3h), consistent with its comparatively lower interface adhesive strength. As interfacial strength approaches or exceeds the cohesive strength of the polymerized matrix and the adjacent bone substrate, the fraction of CF increases,^32^ and measured strength rise accordingly. Mixed failure represents a transitional regime where part of the interface debonds and the remaining load transfers into the bulk, producing concurrent interfacial and cohesive fracture features. Accordingly, AF fraction correlates negatively with adhesive strength, CF fraction correlates positively, and MF varies around intermediate strengths. Scanning electron microscopy and micro-computed tomography imaging confirmed close adaptation of TNC to both cortical and cancellous bone surfaces (Figs. 3k, S7). From the above images, it can be speculated that the absence of AF in cancellous bone samples might be attributed to the interlocking between the adhesive and the bone’s porous microstructure.^33^

### In vitro and in vivo biocompatibility

**Fig. 4:**
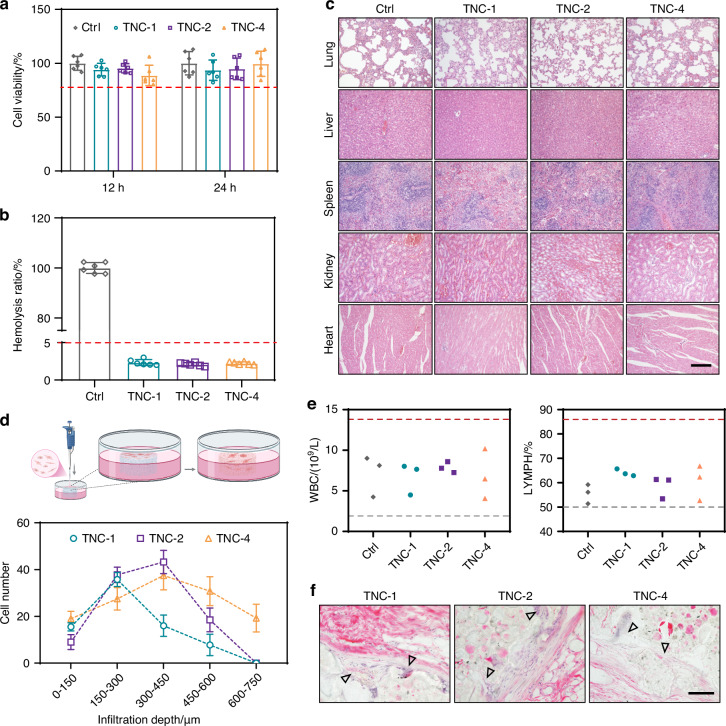
Biocompatibility of TNC. **a** Cell viability determined by co-incubation of MC-3T3 cells on the TNC surface for 12 and 24 h (*n* = 6). The red dotted line represents the threshold of 80% cell viability. **b** Hemolysis ratio of TNC (*n* = 6). Triton X-100 was set as the positive control. The red dotted line represents 5% hemolysis ratio. **c** The representative H&E staining of the main organs after the TNC subcutaneous implanted for 2 weeks. Scale bar: 200 μm. **d** Schematic illustration of cell infiltration and cell numbers in various depth after 72 h co-incubation of MC-3T3 cells and the TNC. Analysis was performed with confocal laser scanning microscopy z-stacking (*n* = 4). **e** White blood cell (WBC) and lymphocyte (LYMPH) number of rat tail vein after TNC subcutaneous implanted for 24 h (*n* = 3). The gray dotted line represents lower limit of normal, and the red dotted line represents the high limit of normal. **f** Hematoxylin-eosin staining analysis of TNC implanted subcutaneous for 14 days. The black triangle represents tissues infiltrated into the TNC. Scale bar: 100 μm

Biocompatibility is a fundamental requirement for bone adhesives.^34–36^ Direct co-culture of MC-3T3 cells with TNC demonstrated cell viability exceeding 80% in all groups, with no significant difference compared to the control group (Fig. 4a). Hemocompatibility was also evaluated because of the inevitable exposure to blood during clinical application.^37,38^ TNC-1, TNC-2, and TNC-4 exhibited hemolysis ratios below 5%; the observation is indicative of minimal hemolytic activity (Fig. 4b). These outcomes confirm the favorable cytocompatibility of TNC under in vitro conditions.

In vivo toxicity was evaluated through subcutaneous implantation of TNC in rats. Hematoxylin and eosin (H&E) staining of the lung, liver, spleen, kidney, and heart revealed no pathological abnormalities when compared with untreated controls (Fig. 4c). Routine blood parameters remained within normal limits, further supporting systemic biocompatibility (Figs. 4e and S8). These data affirm the absence of acute toxicity and support the in vivo use of TNC.

Cell migration is essential for effective bone repair. The extent of cellular and tissue infiltration into TNC was examined to assess biointegration. Significant penetration of MC-3T3 cells into the interior of TNC was observed (Fig S9). In both TNC-1 and TNC-2, cell infiltration reached depths of approximately 550 μm. Although infiltration depths were similar, TNC-l exhibited fewer cells in deeper regions (>300 μm), indicative of relatively lower permeability.^15^ In contrast, TNC-4 supported cell migration up to 750 μm in depth (Figs. 4d and S9). The enhanced infiltration is likely attributable to the larger pore size and higher porosity of TNC-4, as these factors are known to facilitate bone regeneration.^15^

In vivo infiltration was also evaluated after 14 days of subcutaneous implantation. All TNC formulations demonstrated favorable histocompatibility (Figs. 4f and S10). Staining with H&E confirmed the presence of host cells and connective tissue within internal pores (Fig. 4f). The observation confirms that TNC supports tissue integration and provides a microenvironment that is conducive to cellular migration.

### Degradation behavior

**Fig. 5:**
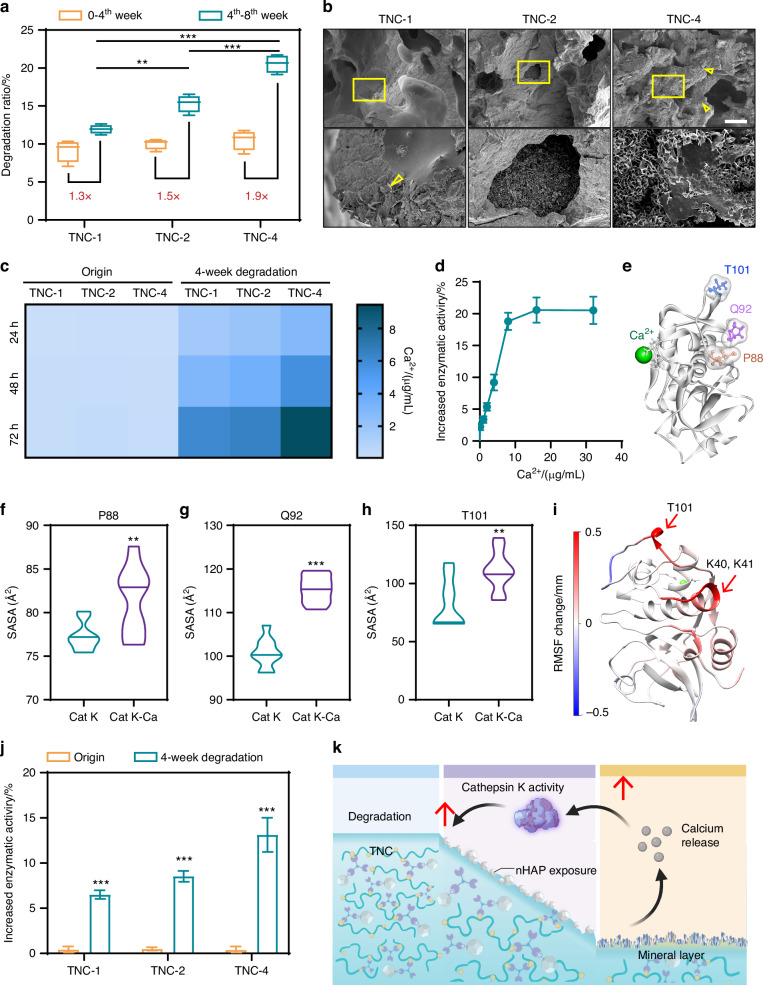
Degradation performance of TNC. **a** Degradation ratio of TNC in cathepsin K for 0–4^th^ week and 4^th^–8^th^ week (*n* = 4), whiskers extending to 1.5×IQR boundaries. **b** Representative SEM images of TNC degraded in cathepsin K for 4 weeks. The yellow triangle represents the new-formed mineral crystals. Scale bar: 100 μm. The image in the yellow square was further manifested. **c** The calcium release property of TNC before and after 4-week degradation (*n* = 4). **d** The increased enzymatic activity of cathepsin K under different concentration of calcium ions. **e** The position of residues P88, Q92 and T101 in the structure of cathepsin K. The solvent accessible surface area of residues P88 (**f**), Q92 (**g**) and T101 (**h**) in cathepsin K, before and after its binding with calcium ion. **i** The root-mean-square fluctuation change of cathepsin K structure after combined with calcium ion. **j** Cathepsin K activity influenced by TNC before and after 4-week degradation (*n* = 4). **k** Schematic illustrating the interaction between TNC degradation and cathepsin K. **P < 0.01; ***P < 0.001

An ideal bone adhesive should exhibit a high degradation rate during the bone remodeling phase.^39^ Cathepsin K is secreted by osteoclasts during fracture healing. This enzyme plays a central role in callus resorption and bone remodeling.^40,41^ To determine degradation responsiveness, TNC was subjected to enzymatic conditions involving cathepsin K. Degradation of TNC was significantly accelerated after four weeks (Fig. 5a). Between weeks 4 and 8, TNC-4 demonstrated a 1.9-fold increase in degradation rate compared to the initial four-week period. Incidentally, TNC-4 exhibited a higher overall degradation ratio than TNC-1 and TNC-2 at the same time-point (Fig. S11).

A mineral layer was observed on the surface of TNC after four weeks of degradation (Fig. 5b). Energy-dispersive X-ray spectroscopy analysis revealed elevated levels of calcium (Ca) and phosphorus (P), confirming the presence of calcium phosphate (CaP)-based minerals (Fig. S12a). As shown in Fig. S12b, after 4 weeks of degradation, the XRD patterns of the sample showed a reduction in the HAP diffraction peaks compared with those of the original one. Besides, it is accompanied by the enhancement and broadening of the peak at 2θ ≈ 45.5°, which may suggest the formation of β-TCP during this process (JCPDS #09-0169). Collectively, these findings suggest the occurrence of a dissolution-reprecipitation process of nHAP during TNC degradation, which is likely a key mechanism responsible for the formation of a crystalline mineral layer on the material surface. The extent of mineralization varied by formulation, with TNC-4 displaying more extensive mineral coverage compared to TNC-1 and TNC-2. This outcome is due to the higher collagen content in TNC-4. The increased collagen content accelerates degradation of organic components and exposes nano-hydroxyapatite, which then promotes mineral recrystallization.

The mineralized TNC released significantly more Ca²⁺ than its original form (Fig. 5c). Calcium ions were found to upregulate cathepsin K activity in a dose-dependent manner among 0.1–8 μg/mL (Fig. 5d). The possible mechanisms were as follows. The structure of cathepsin K contains calcium-binding sites (N40, E59, Q73), which modulate its enzymatic behavior.^14,16,42^ Binding of Ca²⁺ induces conformational changes in cathepsin K, increasing the flexibility and exposure of several functional residues (Fig. 5e–i). Residues P88 and T101 are involved in dimer formation, which is essential for collagen cleavage. K40 and K41 enhance interaction with glycosaminoglycans and contribute to collagenase activity. Exposure of Q92 facilitates collagen unfolding.^14,42^ These changes collectively promote cathepsin K activity.

A marked increase in cathepsin K activity was observed after co-culture with degraded TNC. In contrast, no such effect occurred with undegraded TNC (Fig. 5j). This enhancement is likely attributable to Ca²⁺ released from the mineral layer. Among the three groups, TNC-4 released the highest amount of Ca²⁺ and correspondingly induced the strongest enzymatic activity. The observation was well aligned with the higher degradation rate of TNC-4 after four weeks.

These findings indicate that TNC is degradable by cathepsin K and that the degradation rate of TNC increases after an initial phase (Fig. 5k). This adaptive degradation behavior corresponds well with the bone remodeling timeline, supporting the clinical potential of TNC as a resorbable bone adhesive. Moreover, the degraded TNC can stimulate osteoclasts to secrete cathepsin K (Fig. S13), which further accelerates its degradation rate in the bone remodeling phase. Then, the osteoclast cultures were exposed to increasing Ca²⁺ concentrations, and cathepsin K in the supernatant was quantified by ELISA. As shown in Fig. S14, cathepsin K release rose with Ca²⁺ from 0–8 μg/mL, with a decline beyond 8 μg/mL, demonstrating a dose-dependent but non-linear response to calcium.

### Osteogenic differentiation

Alizarin red S staining was used to evaluate the osteogenic differentiation of MC-3T3 cells after 21 days of co-culture with TNC. Compared with the control group, all TNC formulations promoted greater calcium nodule deposition (Fig. S15). The enhanced mineralization is likely attributed to the presence of nano-hydroxyapatite crystallites within the experimental bone adhesive.^43,44^ Among the tested groups, TNC-4 induced the highest level of mineral deposition.

Similar trends were observed in alkaline phosphatase activity, as well as the expression levels of osteogenic proteins such as osteocalcin and type I collagen (Fig. S15). The differences in osteogenic potential among the formulations are likely related to a combination of material properties. Briefly, higher open porosity increases accessible surface and transport pathways, which facilitates protein adsorption, cell infiltration, and subsequent matrix deposition.^45^ Greater hydrophilicity lowers the contact angle and promotes the rapid formation of a cell-adhesive provisional protein layer, improving early attachment and spreading.^46^ Formulations that degrade faster expose collagen domains sooner and refresh the surface chemistry, while also avoiding long-term physical shielding of the repair site. Finally, nano-hydroxyapatite–derived calcium and phosphate can modulate osteogenic signaling and mineral deposition^47^; the timing and magnitude of ion release depend on both crosslink density and water uptake. Collectively, these properties underlie the superior osteogenic differentiation potential demonstrated by TNC-4.

### Fracture healing property of TNC

Based on its strong adhesive strength, controlled degradation, and osteogenic potential, TNC-4 was selected for in vivo testing of fracture repair. A modified adhesive (TC-4) was also prepared. The modified bone adhesive shares the same tri-HDI and collagen ratio as TNC-4 but lacks nano-hydroxyapatite (Fig. S16).

**Fig. 6:**
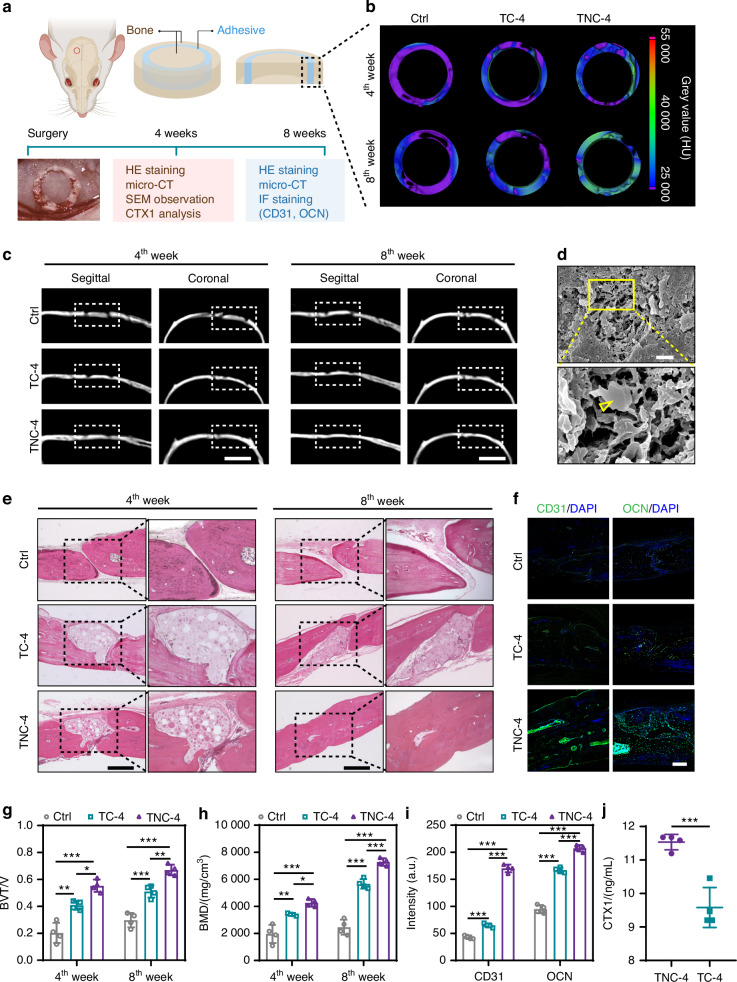
Bone fracture healing with TNC-4 in vivo. **a** Schematic of rat skull fracture. A 4 mm diameter of circle skull fracture was fixed with experimental bone adhesives. Representative micro-computed tomography image (**c**) and gray value in the fracture area (**b**) on the 4^th^ and 8^th^ week after surgery, scale bar: 4 mm. **d** Mineral crystal formed in TNC-4 after skull healing for 4 weeks, scale bar: 1 µm. The area in yellow square was further illustrated. The yellow triangle refers to the mineral crystallites. **e** Hematoxylin-eosin staining of the fracture on the 4^th^ and 8^th^ week after surgery, scale bar: 500 µm. The area in black dotted square was further manifested. Representative immunofluorescence images (**f**) and quantitative analysis (**i**) of CD31 and osteocalcin (OCN) after fracture healed for 8 weeks, scale bar: 200 µm. Quantitative analysis of (**g**) ratio of bone volume/total volume (BV/TV) and (**h**) bone mineral density by micro-CT (*n* = 4). **j** Concentration of C-terminal peptide of type I collagen (CTX1) after fracture healed for 4 weeks (*n* = 4). *P < 0.05; **P < 0.01; ***P < 0.001

A skull fracture model was used to examine the ability of the adhesive to stabilize free bone fragments. Bone segments were treated with TNC-4, TC-4, or left untreated (Fig. 6a). Healing was evaluated at 4 and 8 weeks after surgery. In the untreated group, micro-computed tomography revealed persistent dislocation of the circular bone fragment at both time points (Fig. 6b, c). Spontaneous healing was not observed in the absence of fixation.

In contrast, TNC-4 achieved complete integration between the fragment and the surrounding skull bone. After 8 weeks, the fracture site appeared well stabilized (Fig. S17). Quantitative analysis showed that the bone volume to tissue volume ratio (BV/TV) was significantly higher in the TNC-4 group (0.67 ± 0.04) than in TC-4 (0.51 ± 0.05) and the control group (0.29 ± 0.05) (Fig. 6g). Bone mineral density followed a similar pattern. Values recorded were 2 459 ± 555 mg/cm³ for TNC-4, 5 666 ± 351 mg/cm³ for TC-4, and 7 281 ± 274 mg/cm³ for the control group (Fig. 6h). These results suggest that TNC-4 promotes effective integration of bone fragments.

Histological examination supported the micro-computed tomography findings. At week 8, the fracture site in the TNC-4 group was bridged primarily by woven bone (Fig. 6e). An elevated expression of osteocalcin (OCN) was detected, suggesting active osteogenesis (Fig. 6f, i). Given that delayed degradation of bone repair materials can impede vascularization and hinder bone healing, the vascular ingrowth was further assessed.^48,49^ Immunostaining revealed enhanced CD31 expression in the TNC-4 group (Fig. 6f, i), which was likely attributable to the appropriate degradation rate of TNC-4. This timely degradation appeared to create sufficient space to facilitate both vascularization and new bone deposition. Consequently, the majority of the TNC-4 was replaced by newly formed bone, with only minimal residues detectable. In contrast, TC-4 exhibited limited degradation over the same period, with much of the material remaining (Fig. 6e).

The TNC-4 also led to higher cathepsin K activity than TC-4 in vivo. The serum level of C-terminal telopeptide of type I collagen (CTX1) reflects this activity.^50,51^ At 4 weeks, CTX1 levels were higher in the TNC-4 group compared to TC-4 (Fig. 6j). The enhanced activation of cathepsin K accelerated polyurethane degradation and led to more exposure of collagen in TNC-4 (Fig. S18). Collagen is highly degradable, which further widening the degradation rate difference between TNC-4 and TC-4. Besides, the TNC-4 group exhibited a larger positive area of Tartrate-Resistant Acid Phosphatase (TRAP) staining (Fig. S19), suggestive of enhanced osteoblast activity. It’s predominantly localized around the adhesive. Notably, the absolute TRAP-positive proportion remained small and fracture healing appeared satisfactory, with no histologic signs of abnormal resorption. The result indicates that cathepsin K activity associated with TNC degradation is controlled rather than deleterious.

A newly formed mineral layer was also observed in TNC-4 in vivo (Figs. 6d, S20). This observation is consistent with earlier findings and may contribute to increased cathepsin K activation. Taken together, these results confirm that TNC-4 supports stable bone fixation, promotes bone regeneration, and undergoes timely degradation. These highly desirable properties render TNC-4 a potential candidate for fracture repair.

## Discussion

An effective bone adhesive should exhibit suitable curing time, strong adhesive strength and excellent biological performance.^7–9^ For optimal biological performance, the material is expected to demonstrate effective osteogenic activity, biocompatibility, and appropriate degradation behavior. Previous studies have made significant progress in improving adhesive strength, biocompatibility, and osteogenic capability.^5,6^ However, achieving appropriate degradation behavior for bone adhesive still remains a challenge. An ideal bone adhesive should exhibit a slow degradation rate during the initial healing phase to maintain fragment stability, while accelerating degradation during the remodeling phase to facilitate bone healing.^10^

To address the aforementioned challenges, this study developed a tri-HDI-bridged collagen/nHAP polyurethane that couples surgical handling (fast cure, wet adhesion) with biologically timed, Ca²⁺-potentiated enzymatic resorption. First, collagen was incorporated as a soft segment of the polyurethane, leveraging the rapid reaction between the amino groups of collagens and -NCO groups of tri-HDI at room temperature.^52^ This enables the bone adhesive to cure within 10 minutes, meeting the required setting time for clinical applications.^10^ Moreover, collagen exhibits excellent biocompatibility, which contributes to enhancing the safety of the polyurethane-based material.

Besides, the TNC exhibits strong initial adhesion to both cortical and cancellous bone, even under wet or blood-contaminated conditions, making it highly suitable for clinical applications. The possible adhesive mechanism is as follows. Adhesion strength arises from internal cohesion within the adhesive, and interfacial bonding with the substrate.^53^ A schematic of the proposed adhesion mechanism is provided in Fig. 3l. Internal cohesion is primarily driven by covalent bonds formed through urea and urethane linkages,^54,55^ as evidenced by the high mechanical strength of cured samples. Interfacial adhesion is enabled by chemical reactions between isocyanate (-NCO) groups in TNC and hydroxyl or amine groups present in bone tissue.^56,57^ In addition, hydrogen bonding at the interface is facilitated by the shared presence of amine and hydroxyl groups in both the adhesive and bone surface.^58,59^

It’s worth noting that the collagen content affected the properties of TNC. The absorption of ambient moisture during TNC curing served as a foaming agent, which produced a porous polyurethane matrix structure.^19^ Additionally, the air incorporation during collagen mixing may account for the observed increase in pore structure with higher collagen concentrations. The increased pore structure led to mechanical strength reduction and cell infiltration improvement, as reported in previous studies.^20,21^ This suggests that the TNC properties can be adjusted to match the requirements of diverse application scenarios by mixing collagen content at different ratios.

Moreover, TNC exhibits temporally-synchronized degradation with the stages of bone healing as expected. At the initial stage, TNC exhibits a slow degradation rate, to provide essential mechanical support for stabilizing bone fragments. During degradation in the presence of cathepsin K, who is the key enzyme during fracture healing,^40,41^ a mineral layer formed on the surface of TNC. Formation of this mineral layer is likely due to the incorporation of nano-hydroxyapatite. Previous studies have shown that nano-hydroxyapatite-containing adhesives produce mineral layers upon exposure.^10,15^ The mineral layer might be resulted from a dissolution-reprecipitation process of nHAP.^60^ This layer enhanced the release of calcium ions from the degraded material. The cathepsin K contains calcium-binding sites, and the released Ca²⁺ further activated cathepsin K enzymatic behavior by modulating its structure.^14,16^ Besides, the degraded TNC can also stimulate osteoclasts to secrete cathepsin K, which may be associated with the activation of osteoclast activity by the released calcium ions.^61^ As a result, TNC exhibited a higher degradation rate during the later stages of bone healing. This timely degradation feather could create sufficient space to facilitate both vascularization and supports effective bone regeneration. In vivo testing further confirmed that TNC promoted stable bone bridging and accelerated healing.

Despite these promising results, several limitations warrant further investigation. The detailed mechanism of mineral layer formation during TNC degradation still needs clarification and requires more in-depth characterization. Detailed characterization of TNC’s degradation behavior in vivo is still needed. Additionally, while TNC demonstrated excellent biocompatibility and osteogenic potential, optimizing the balance between adhesive strength and degradation rate is crucial for clinical applications. Future studies should also explore the performance of TNC in other fracture models, such as long bone fractures, to fully assess its clinical potential.

In conclusion, this study presents a novel bio-responsive bone adhesive that synchronizes its degradation with bone healing stages. The unique properties of TNC, including its room-temperature cure, strong adhesive strength, controlled degradation, and osteogenic potential, make it a promising material for fracture fixation and bone regeneration. The design concept establishes a clinically relevant framework for next generation materials that actively synchronize with bone regeneration through a bio-responsive way. To further enhance biological interactions during adhesive degradation and bone remodeling, bioactive components such as RGD peptides can be integrated into the adhesive system. These components are intended to improve osteoclast recruitment and promote bone formation by mimicking endogenous biological signals.^62^ In addition, due to the diversity of fracture sites, and fracture patterns, the next-generation bone adhesives must also accommodate the personalized fracture geometry and mechanical demands of diverse patients.^63,64^ Further optimization and evaluation in more complex fracture models will be necessary to translate this technology into clinical practice.

## Materials and methods

### Materials

Tri-HDI (NONE7976, Macklin, Shanghai, China), and nano-hydroxyapatite (H861730, Macklin Biochemical Technology Co., Ltd., Shanghai, China) were obtained from Macklin. Ditin-butyl dilaurate (CF701522, Merck KGaA, Darmstadt, Germany), acetyl chloride (114189, Merck KGaA, Darmstadt, Germany), type I collagen (C7661, MilliporeSigma, Burlington, MA, USA), cathepsin K (HY-P72156A, MedChemExpress, Shanghai, China), and MC-3T3 cells (CL-0378, Procell Life Science & Technology Co., Ltd., Wuhan, China) were used for subsequent experiments.

Laboratory rats were obtained from the Laboratory Animal Research Center of the Fourth Military Medical University, Xi’an, China. The rats were maintained in pathogen-free conditions. All animal procedures followed protocols approved by the Institutional Animal Care and Use Committee of the Fourth Military Medical University (IACUC-2023-kq-048). The procedures complied with the guidelines of the National Institutes of Health for the care and use of laboratory animals. The rats were anesthetized via intraperitoneal injection of sodium pentobarbital at a dose of 30 mg/kg, and sacrificed by intraperitoneal injection of excessive pentobarbital sodium. The experimental scheme is illustrated in Fig. S21.

### Preparation of TNC

Tri-HDI (0.50 g, 1.15 mmoL) and acetyl chloride were mixed with nHAP (0.33 g, 657 μmoL) at 75 °C under a nitrogen atmosphere to obtain pre-TNC. Ditin-butyl dilaurate was used as the catalyst in this reaction. The resulting pre-TNC was then combined with type I collagen at varying mass ratios to form TNC. Chemical titration showed that a 1% collagen mass ratio was the minimum required to fully consume the isocyanate (-NCO) groups. Based on this result, TNC formulations containing 1%, 2%, and 4% collagen were prepared and labeled as TNC-1, TNC-2, and TNC-4, respectively. Besides, tri-HDI was directly mixed with 4% collagen under the same reaction conditions but without nHAP. This material was designated as TC-4.

### Characterization

Thermal images during polymerization were recorded every 10 s using a thermal infrared camera (HT-19, HTI Instrument, Guangdong, China). Infrared spectra were obtained with an ATR-FTIR spectrometer (BRUKER Vertex 70, Bruker Corporation, Billerica, MA, USA) and normalized as previously described.^37^

Elemental composition of TNC was analyzed using X-ray photoelectron spectroscopy (XPS; K-Alpha, Thermo Fisher Scientific, Waltham, MA, USA). The C 1 s region of the XPS spectra was further examined for detailed analysis. X-ray diffraction patterns were collected at room temperature over a 2θ range of 10°–60° using a diffractometer (SmartLab, Rigaku, Japan).

Morphology and surface elemental distribution were characterized using scanning electron microscopy (SEM; S-4800, Hitachi, Tokyo, Japan). Porosity and pore size were quantified using ImageJ software (National Institutes of Health, Bethesda, MD, USA).

Thermal stability was evaluated with a dynamic thermomechanical analyzer (Bruker Avance Neo 400WB, Rheinstetten, Germany). Samples were heated from 30 °C to 700 °C at a rate of 20 °C /min.

### Mechanical properties

The TNC specimens were polymerized at room temperature for 1 h in a Teflon mold with a sample size of 5 mm in diameter and 3 mm in height. Compressive modulus was then measured using a universal testing machine (Shimadzu GS-X10KN, Japan) at a loading rate of 0.5 mm/min.

For tensile testing, TNC was cured in a dumbbell-shaped mold (50 × 4 × 2 mm³) for 1 h. Testing followed the standard tensile test method and was performed using the same machine at a constant speed of 1 mm/min.^10^

The water contact angle of TNC was measured using a contact angle goniometer (Kruss Co Easy Drop K100, KRÜSS Scientific, Hamburg, Germany) with a droplet pumping rate of 2.00 μL/s.^65^

Water absorption and expansion rates were determined by comparing mass and volume before and after immersion in water for 30 min, following previously described procedures.^38^

### Adhesive performance

Fresh cancellous and cortical bovine bone was obtained from the abattoir and cut into segments measuring 3 × 6 × 20 mm³. A 10 μL volume of TNC was applied to bond the bone specimens, creating an adherent area of 36 mm². Specimens were assembled in either a lap shear or butt joint configuration to determine shear and tensile strength, respectively. Bonding was conducted under three conditions: dry, submerged in simulated body fluid, or immersed in blood, all at room temperature for 30 min. Bonded specimens were then tested using a 5 kg load and an AGS-X universal testing machine (Shimadzu, Kyoto, Japan) at a constant tensile speed of 1 mm/min. Adhesive strength was determined by dividing the maximum load by the bonding area. Fracture modes were recorded and examined using SEM and micro-computed tomography (Inveon, Siemens Preclinical, Knoxville, TN, USA). A rat femur fracture model was also established by transecting the mid-diaphysis. The fractured ends were bonded with TNC for in vivo evaluation.

### In vitro biocompatibility

The TNC leaching liquid was prepared by immersing the material in alpha-Eagle’s Minimum Essential Medium (α-MEM, PM150421, Pricella, Wuhan, China) at a concentration of 100 mg/mL for 48 h. The MC-3T3 cells (3 × 10³ cells per well) were cultured in this leaching liquid for 12 and 24 h in an incubator, with 10% fetal bovine serum and 5% penicillin-streptomycin added. Cytotoxicity was evaluated using the CCK-8 assay (CCK-8, CK04, Dojindo Molecular Technologies, Rockville, MD, USA) in 96-well plates.

For hemocompatibility evaluation, a red blood cell suspension was prepared from fresh rat blood and subjected to hemolysis testing as previously described.^37^ To examine cellular adhesion and infiltration, MC-3T3 cells were seeded uniformly onto the surface of TNC in 6-well plates at a concentration of 1 × 10⁴ cells/mL. After 72 h of co-culture, the cells were stained using a calcein/propidium iodide cell viability kit (C2015M, Beyotime, Shanghai, China).^15^ Cell migration depth and distribution within TNC samples were visualized by laser scanning confocal microscopy (Nikon A1R, Nikon Corporation, Minato-ku, Tokyo, Japan) with 3D reconstruction.

### In vivo biocompatibility

The TNC specimens (5 mm in diameter and 3 mm in height) were implanted subcutaneously in the backs of rats (six-week-old, Sprague-Dawley rats). A sham-operated group served as the control. After 24 h, blood was collected from the inner canthus using capillary tubes, and routine blood analysis was performed with an automated analyzer (Sysmex, Kobe, Japan). On day 14, the heart, liver, spleen, lungs, and kidneys were excised for histological processing and staining. At the same time, the TNC specimens and surrounding tissues were harvested. All excised samples were fixed, embedded, and subjected to hematoxylin and eosin (H&E) staining for further histological evaluation.

### Degradation properties

The initial weight of TNC specimens (5 mm in diameter and 3 mm in height) was recorded. Specimens were then incubated in cathepsin K solution (0.25 μg/mL) at 37 °C.^66^ The specimens were collected at 4 and 8 weeks, dried, and weighed to calculate the degradation percentage. For calcium ion release, original and 4-week degraded TNC specimens were immersed in deionized water at an initial concentration of 5 g/mL. After incubation at 37 °C for 24, 48, and 72 h, calcium release was quantified using inductively coupled plasma-mass spectrometry (ICP-MS; NexION 2000, PerkinElmer, MA, USA). Surface morphology of the 4-week degraded TNC was examined by SEM.

### Molecular dynamics simulation

All simulations were performed using the AMBER2024 software package with the AMBER19SB force field and the 12-6 LJ Nonbonded Model. The protein structure of Cathepsin K (PDB ID: 3C9E) was retrieved from the Protein Data Bank. The system was solvated in a TIP3P water box and neutralized with counterions. After energy minimization using the steepest descent algorithm, the system was equilibrated in the canonical ensemble for 100 ps, followed by an isothermal-isobaric ensemble equilibration for another 100 ps. Temperature and pressure were maintained at 300 K and 1 bar, respectively, using the modified Berendsen thermostat and Parrinello-Rahman barostat. A 500-ns production simulation was conducted under periodic boundary conditions, and trajectories were recorded every 50 ps. Root mean square deviation analysis was used to evaluate the stability of the system, while solvent accessible surface area and root mean square fluctuation were calculated to assess structural changes and residue flexibility of Cathepsin K upon Ca²^+^ binding. All trajectory analyses were performed using AMBERTOOLS 2024 (National Institute of Health, Bethesda, MD, USA). Visualizations were generated with Chimera and Visual Molecular Dynamics.

### Cathepsin K activity

To determine cathepsin K activity, the enzyme (200 μg/mL) was incubated with the groups (TNC, 4w-degraded TNC, and Ca^2+^ at 0.1, 1, 2, 4, 8, 16, and 32 μg/mL concentrations) at 37 °C for 24 h. Cathepsin K activity was measured using the method described previously.^67^

### Cathepsin K secretion

Conditioned media were prepared by incubating the samples (TNC-4, 4-week degraded TNC-4, and Ca^2+^ at 0, 4, 8, 16 and 32 μg/ml concentrations) with α-MEM at 37 °C for 24 h. RAW264.7 cells were cultured in 6-well plates and induced to differentiate into osteoclasts using the respective media, as previously reported.^68^ On day 11, multinucleated osteoclast-like cells were observed, indicating successful induction. Culture supernatants were then collected, and the secretion of Cathepsin K was measured using a Mouse CTSK (Cathepsin K) ELISA Kit (E-EL-M0250, Elabscience, Wuhan, China) at 450 nm.

### In vitro osteogenesis

The TNC specimens were co-cultured with MC-3T3 cells (1 × 10⁵ per well) in 6-well plates, followed by osteogenic induction. The medium was replaced with osteogenic induction medium when cell confluency reached 80%.^38^ After 14 days of induction, alkaline phosphatase staining was performed using a commercial kit (C3206, Beyotime, Shanghai, China). The stained samples were observed under a stereomicroscope. Quantification of alkaline phosphatase activity was conducted by calculating grayscale values using ImageJ software.

After 21 days of induction, calcium nodule formation was assessed by Alizarin red S staining (C0148s-2, Beyotime, Shanghai, China). Stained nodules were dissolved in elution solution, and optical density at 560 nm was measured to evaluate mineralization. Standard solutions were prepared following the manufacturer’s instructions.

In parallel, the expression of osteogenesis-related genes influenced by TNC was evaluated using reverse transcription-polymerase chain reaction. On days 7 and 14 of induction, the medium was removed, and total RNA was extracted using the Super FastPure Cell RNA Isolation Kit (Vazyme, Nanjing, China). Reverse transcription was carried out using the PrimeScript RT reagent kit (Takara, Kyoto, Japan), and amplification was performed using real-time fluorescence quantitative PCR (TB Green® Premix Ex Taq™ II, Takara, Kyoto, Japan). Glyceraldehyde-3-phosphate dehydrogenase was used as the housekeeping gene. Expression levels of two osteogenesis-related genes, Type I collagen (Col I) and OCN, were analyzed. Primer sequences are provided in the supplementary information (Table S1).

### In vivo fracture healing

A cranial ring fracture model was established using six-week-old Sprague-Dawley rats. A circular cranial bone fragment (4 mm in diameter) was created with a trephine drill to simulate the fracture. A volume of 20 μL TNC-4 or TC-4 was applied around the edge of the bone fragment. The fragment was then repositioned, ensuring moisture was maintained throughout the procedure. In the control group, the fragment was repositioned without the use of adhesives. The periosteum and muscle layers were closed with 5-0 absorbable sutures. The skin was subsequently sealed with 4-0 non-absorbable sutures.

To examine in vivo cathepsin K activity, serum levels of cross-linked C-telopeptide of type I collagen (CTXI) were quantified at week 4 using a rat CTX I ELISA kit (E-EL-R1456, Elabscience Bionovation Inc., Houston, TX, USA). Rats were euthanized at weeks 4 and 8 after surgery. Cranial samples were collected and fixed in 4% paraformaldehyde. Undecalcified specimens from week 4 were sectioned and analyzed by SEM to determine whether a mineral layer had formed on TNC-4 in vivo.

Decalcified specimens were embedded in paraffin and sectioned for further staining. Histological staining (H&E staining) was performed at both 4 and 8 weeks, while TRAP staining was conducted at week 4, immunofluorescence staining for CD31 and OCN was conducted at week 8. Healing at the surgical site was also evaluated by micro-computed tomography. Grayscale values of the cranial ring-bonded region were analyzed to calculate bone volume fraction (BV/TV) and bone mineral density.

### Statistical analyses

All data were presented as means ± standard deviations. Normality and variance homogeneity of the data were assessed using Shapiro–Wilk and modified Levene’s tests, respectively. Parametric tests (Student’s *t*-test for two groups; one-way analysis of variance with Tukey’s post-hoc test for ≥3 groups) were applied if assumptions were met (*α* = 0.05). Nonparametric alternatives were used otherwise. Analyses were performed in GraphPad Prism 5 package (GraphPad Software, La Jolla, CA, USA).

## Supplementary information

Supplementary information

Video S1

## Data Availability

Data available when requested.
