# Supplementary material for: Bone adhesive with temporally-synchronized degradation for enhanced osteointegration
Source: Bone Res. 2026 Apr 7;14:39. doi: 10.1038/s41413-026-00522-8 (PMC13057037; doi:10.1038/s41413-026-00522-8)
Supplement: Supplementary file 1 — Supplementary information [file 41413_2026_522_MOESM1_ESM.docx]

Supporting Information

Bone adhesive with temporally-synchronized degradation for enhanced osteointegration

Jun-ting Gu^1, 2, #^, Zhi-ting Li^1, #^, Yu-zhu Wang^1, #^, Dong-xiao Hao^1^, Gao-peng Dang^1^, Xiao-Qing Cao^3^, Franklin R. Tay^4^, Ji-hua Chen^1^, Conrado Aparicio^5, 6, *^, Kai Jiao^7, *^, Li-na Niu^1, *^

^#^ *These authors contributed equally to this work*

^1^National Clinical Research Center for Oral Diseases & State Key Laboratory of Military Stomatology & Shaanxi Key Laboratory of Stomatology, School of Stomatology, The Fourth Military Medical University, Xi'an, Shaanxi, 710032, China

^2^National Translational Science Center for Molecular Medicine, Department of Cell Biology, State Key Laboratory of Cancer Biology, The Fourth Military Medical University, Xi’an, Shaanxi, 710032, China

^3^The Third Affiliated Hospital of Xinxiang Medical University, Xinxiang, Henan, 453000, China

^4^Dental College of Georgia, Augusta University, Augusta, GA, 30912, USA

^5^Catalan Institute for Research and Advanced Studies (ICREA), Passeig Luís Companys 23, Barcelona, 08010, Spain

^6^BOBI- Bioinspired Oral Biomaterials and Interfaces, Department of Materials Science and Engineering, EEBE, Technical University of Catalonia (UPC)-Barcelona Tech, C/. Eduard Maristany 16, Barcelona, 08019, Spain

^7^Department of Stomatology, Tangdu hospital; State Key Laboratory of Oral & Maxillofacial Reconstruction and Regeneration & National Clinical Research Center for Oral Diseases & Shaanxi Key Laboratory of Stomatology, School of Stomatology, The Fourth Military Medical University, Xi’an, Shaanxi, 710032, China.

^*^Corresponding author: Li-na Niu, School of Stomatology, The Fourth Military Medical University, Xi’an, China, E-mail: [niulina831013@126.com](mailto:niulina831013@126.com)

Kai Jiao, Department of Stomatology, Tangdu hospital, The Fourth Military Medical University, Xi’an, China, E-mail: [kjiao1@163.com](mailto:kjiao1@163.com)

Conrado Aparicio, Catalan Institute for Research and Advanced Studies (ICREA), Passeig Luís Companys 23, Barcelona, Spain, E-mail: conrado.aparicio@upc.edu

**Figure S1.** The curing process of TNC

**Figure S2.** X-ray photoelectron spectroscopy (XPS) spectra of TNC

**Figure S3.** Representative scanning electron microscope (SEM) images of TNC

**Figure S4.** Thermogravimetric analysis curve of TNC

**Figure S5.** Comparison of the adhesive properties of TNC, commercial adhesives, and previous studies

**Figure S6.** Photographs of bonded bone specimens immersed in simulated body fluid, and blood

**Figure S7.** Representative micro-CT image of the bonding interface.

**Figure S8.** The blood routine examination of rats after TNC subcutaneous implantation for 24 hours

**Figure S9.** Representative images of cell penetration depth in TNC

**Figure S10.** Photographs of the TNC subcutaneous implantation for 14 days

**Figure S11.** Degradation behavior of TNC.

**Figure S12.** Characteristic of the mineral layer formed on TNC after 4-week degradation *in vitro*

**Figure S13.** The osteoclast-derived secretion of cathepsin K influenced by TNC-4.

**Figure S14.** The osteoclast-derived secretion of cathepsin K influenced by calcium ions.

**Figure S15.** Osteogenic inductive property of TNC

**Figure S16.** The characteristic of TC-4

**Figure S17.** The photographs of the skull healed for 8 weeks

**Figure S18**. Representative SEM images of TNC-4 and TC-4 degraded in cathepsin K for 4 weeks

**Figure S19.** Representative TRAP-staining images and quantitative analysis after fracture healed for 4 weeks.

**Figure S20.** SEM-EDX result of mineral layer formed on TNC-4 after 4-week degradation *in vivo.*

**Figure S21.** Flow chart depicting the sequence of experiments conducted in the present study.

**Table S1.** Primer sequences used for RT-PCR.

**Video S1.** The video of lifting a ~5 kg bucket using the TNC-1 glued cortical bone with a 36 mm^2^ bonding area.

**
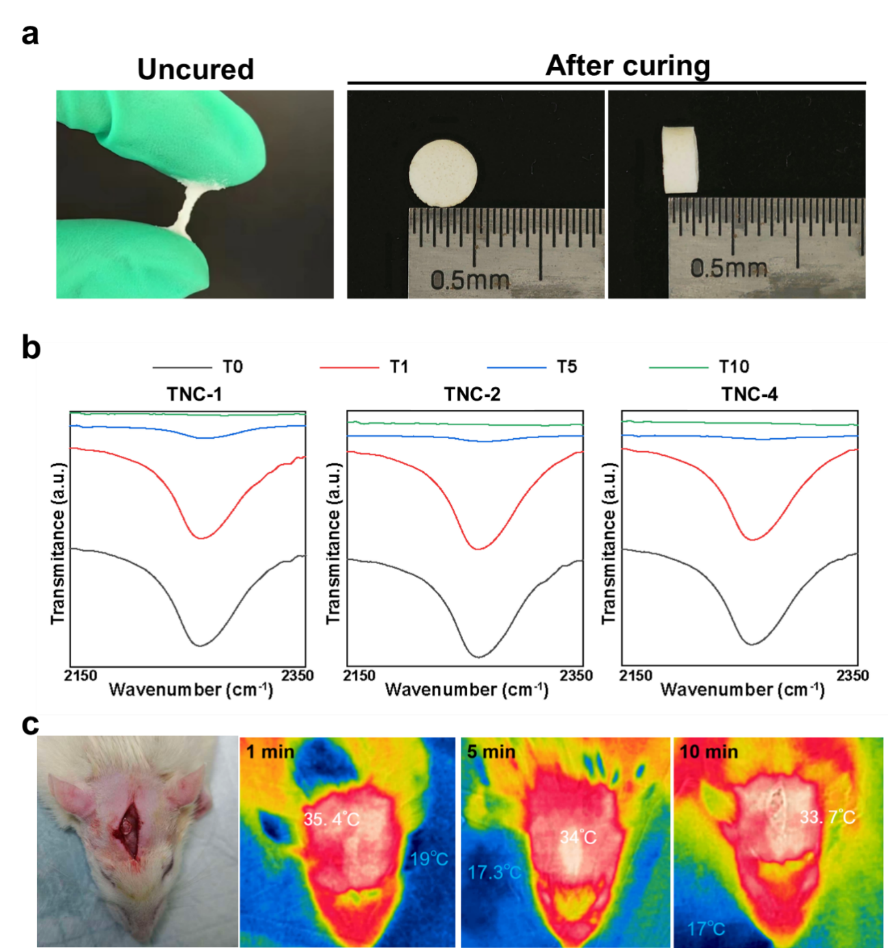
**

**Figure S1.** The curing process of TNC. (a) The representative image of TNC before and after curing. (b) The FTIR spectrum for -NCO peak after TNC cured for a period. (T0, T1, T5, T10 represents for cured for 0, 1, 5, 10 minutes, respectively.). (c) Photograph and thermal images captured during TNC-1 in-situ polymerization in rat cranial defect model.

**
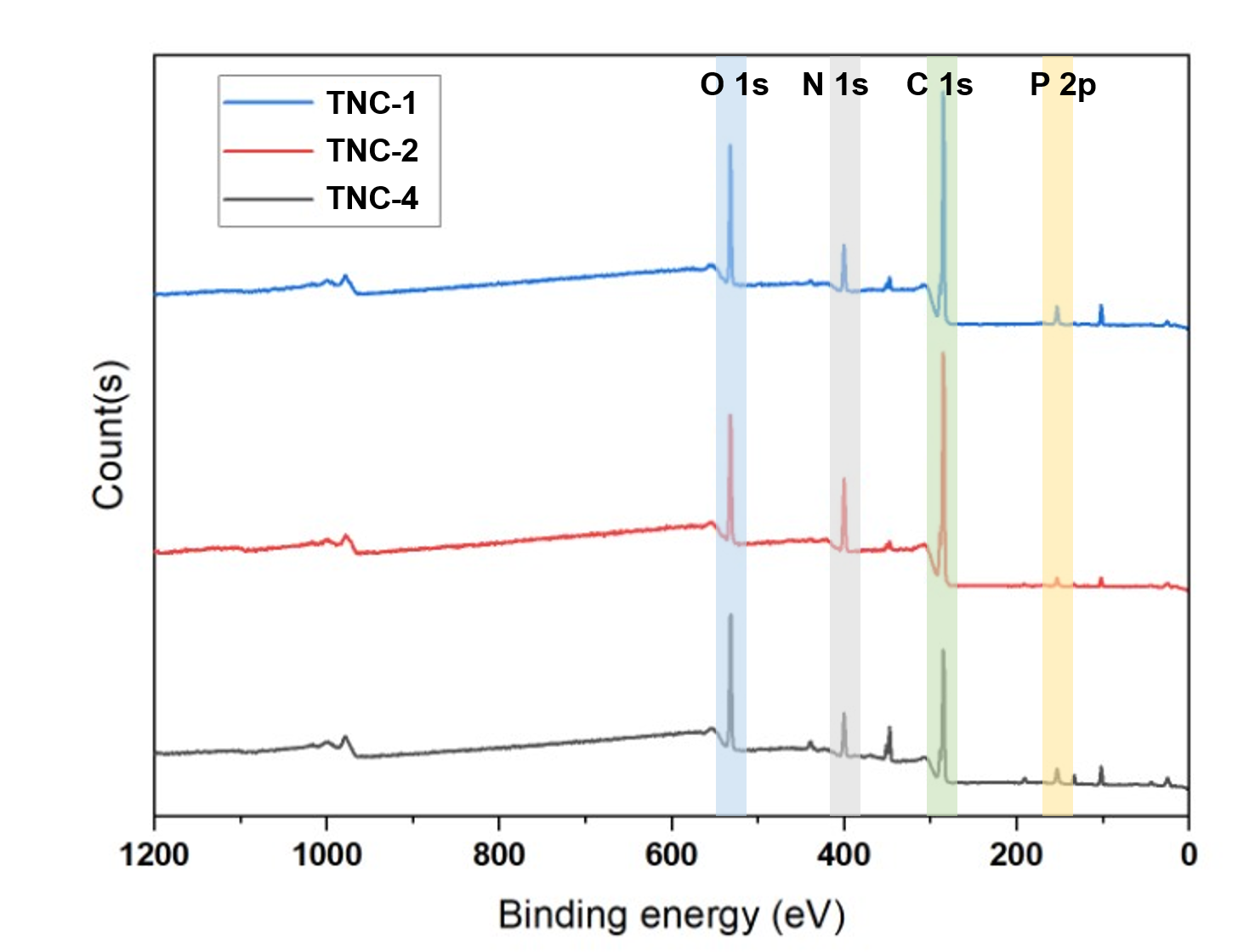
**

**Figure S2.** XPS spectra of TNC.

**
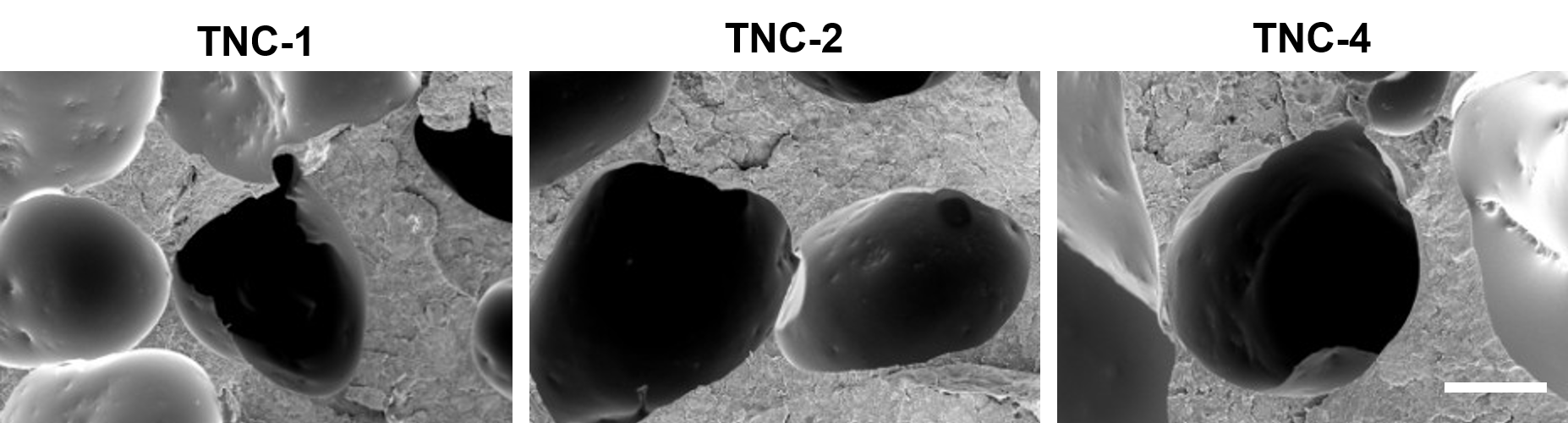
**

**Figure S3.** Representative SEM images of TNC, scale bar: 25 μm.


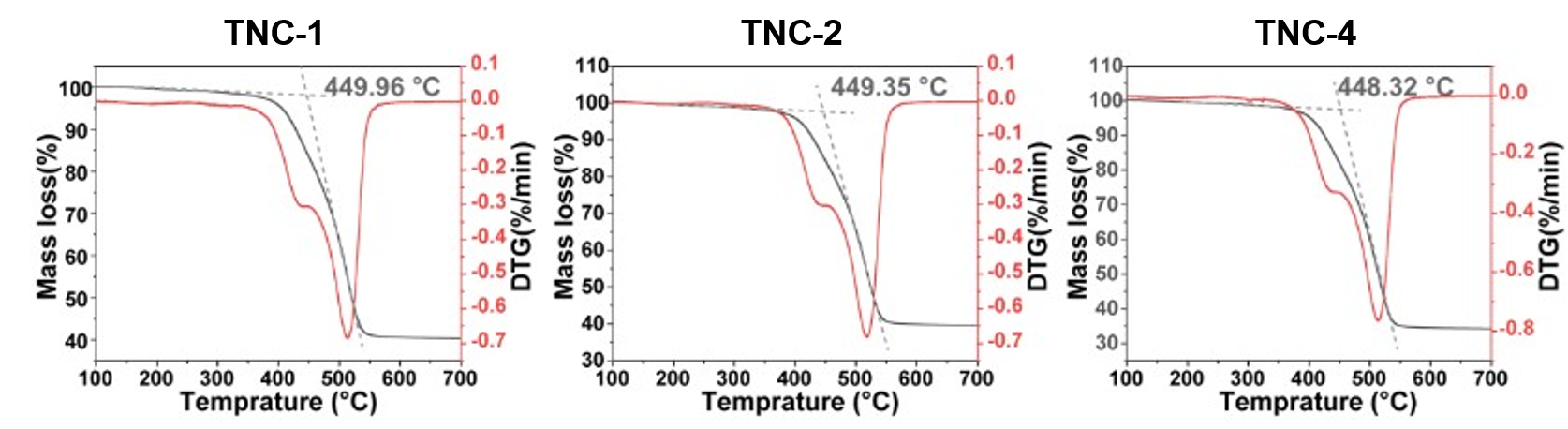


**Figure S4.** Thermogravimetric analysis curve of TNC, heated from 100 °C to 700 °C. DTG: derivative thermogravimetry.


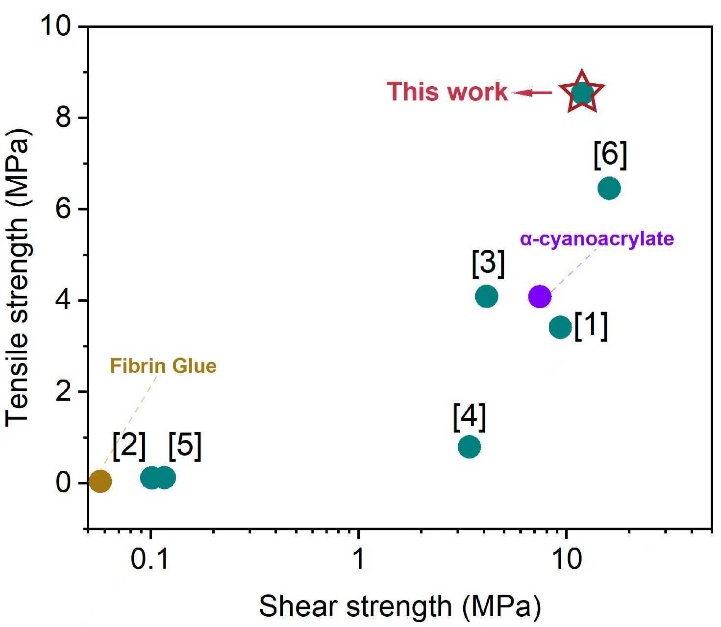


**Figure S5.** Comparison of the adhesive properties of TNC, commercial adhesives (fibrin glue, cyanoacrylate adhesive), and previous studies. ^[1–6]^


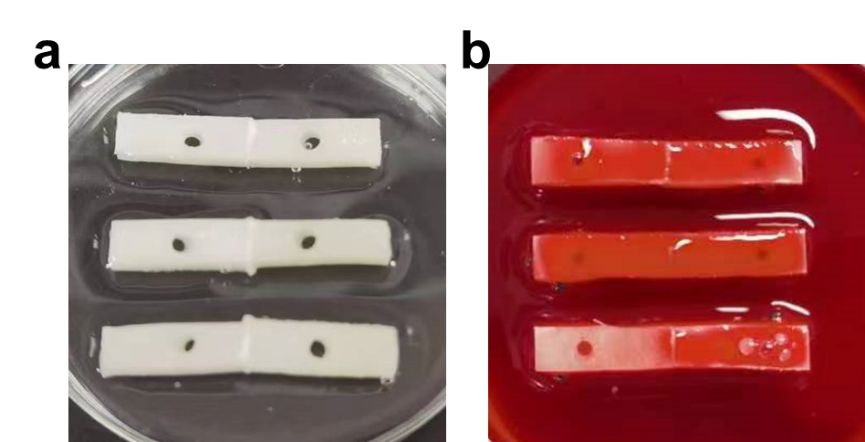


**Figure S6.** Photographs of bonded bone specimens immersed in simulated body fluid (a), and blood (b).


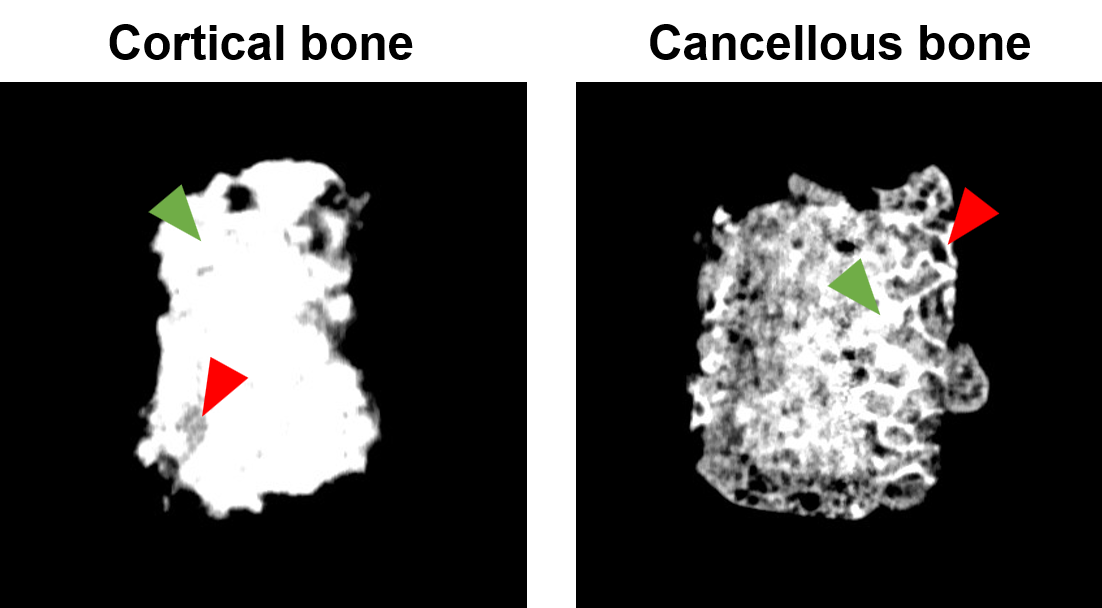
**Figure S7.** Representative micro-CT image of the bonding interface. The green triangle represents the TNC, and the red triangle represents bone tissue.


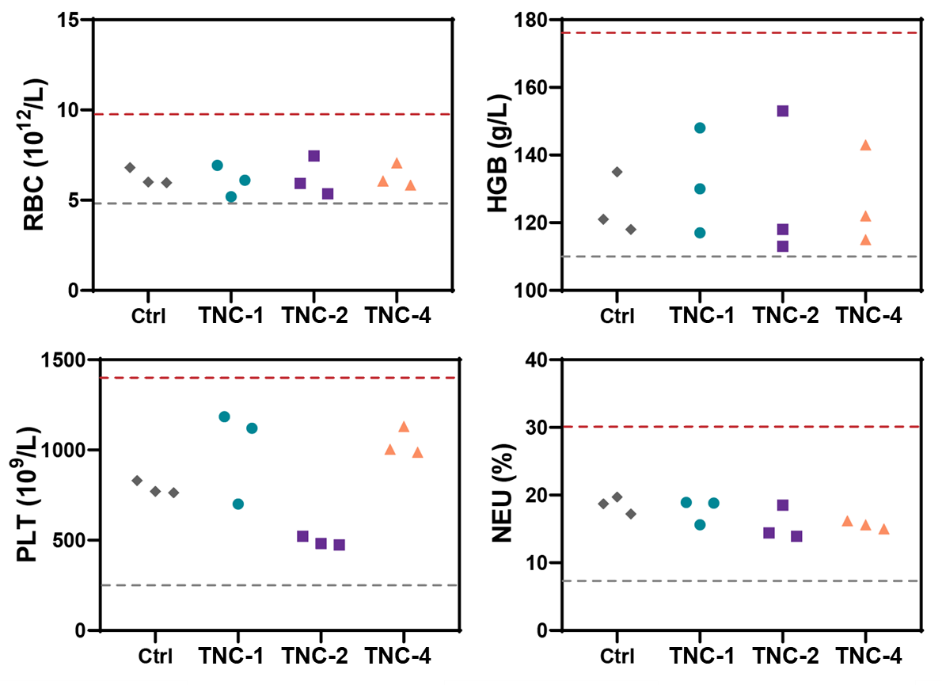


**Figure S8.** The blood routine examination of rats after TNC subcutaneous implantation for 24 h. The gray dotted line represents the lower limit of normal, and the red dotted line represents the upper limit of normal. RBC: red blood cell number, HGB: hemoglobin, PLT: platelet number, NEU: neutrophil percentage.


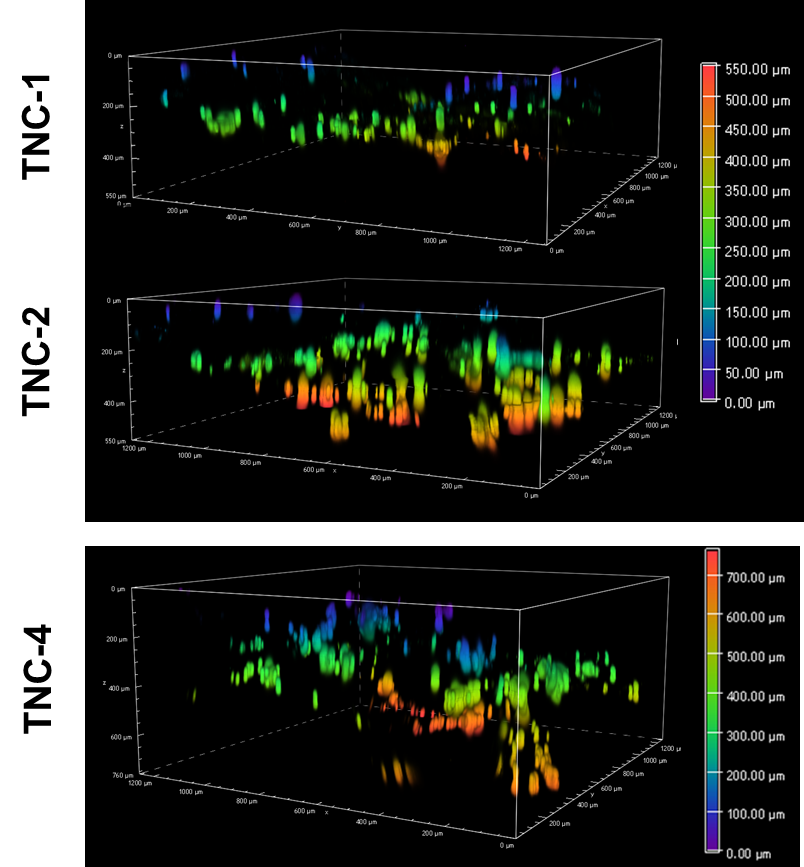


**Figure S9.** Representative images of cell penetration depth in TNC.


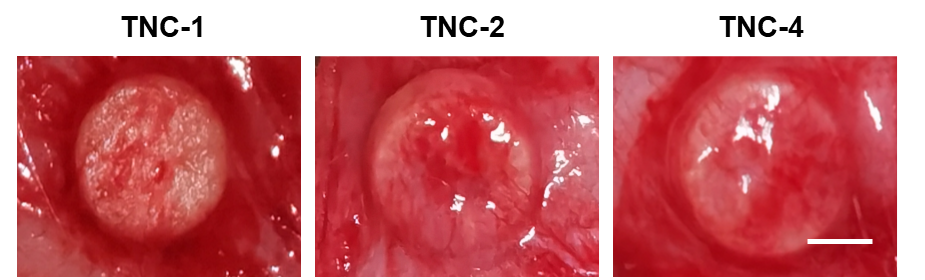


**Figure S10.** Photographs of TNC subcutaneous implantation for 14 days, scale bar: 2 mm.


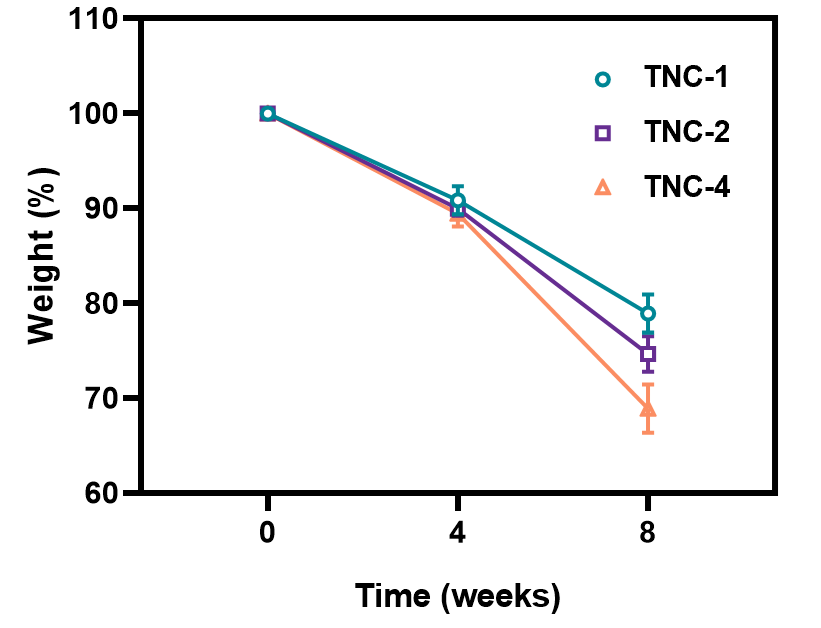


**Figure S11.** Degradation behavior of TNC.


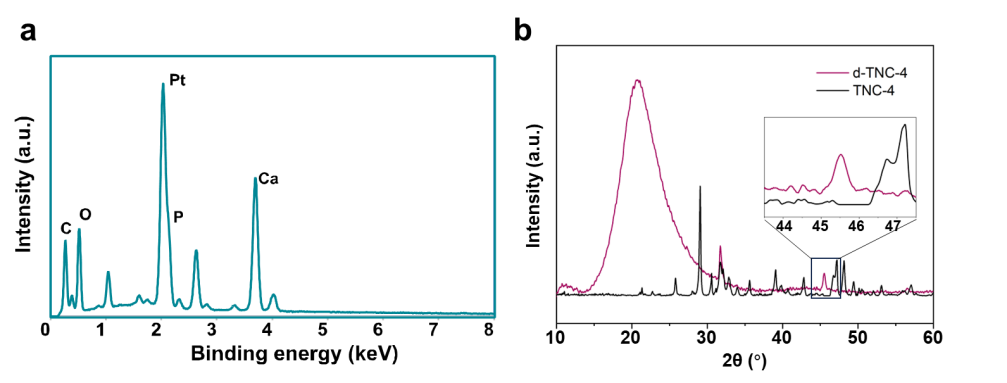


**Figure S12.** Characteristic of the mineral layer formed on TNC after 4-week degradation *in vitro*. (a) SEM-EDX result of the mineral layer. (b) The XRD curve of TNC-4 and d-TNC-4. d-TNC-4: TNC-4 degraded for 4 weeks.


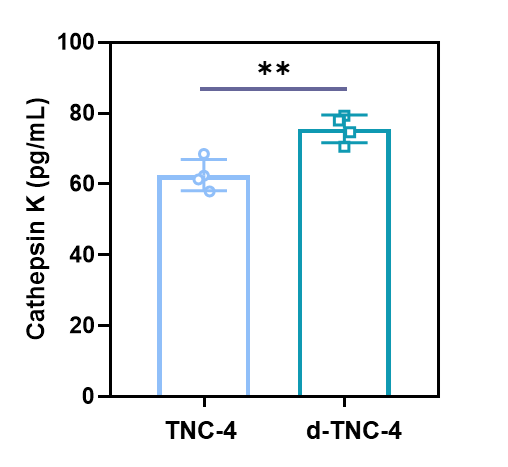


**Figure S13.** The osteoclast-derived secretion of cathepsin K influenced by TNC-4. d-TNC-4: TNC-4 degraded for 4 weeks; **: *p*<0.01.

**Figure S14.** The osteoclast-derived secretion of cathepsin K influenced by calcium ions (n=3).


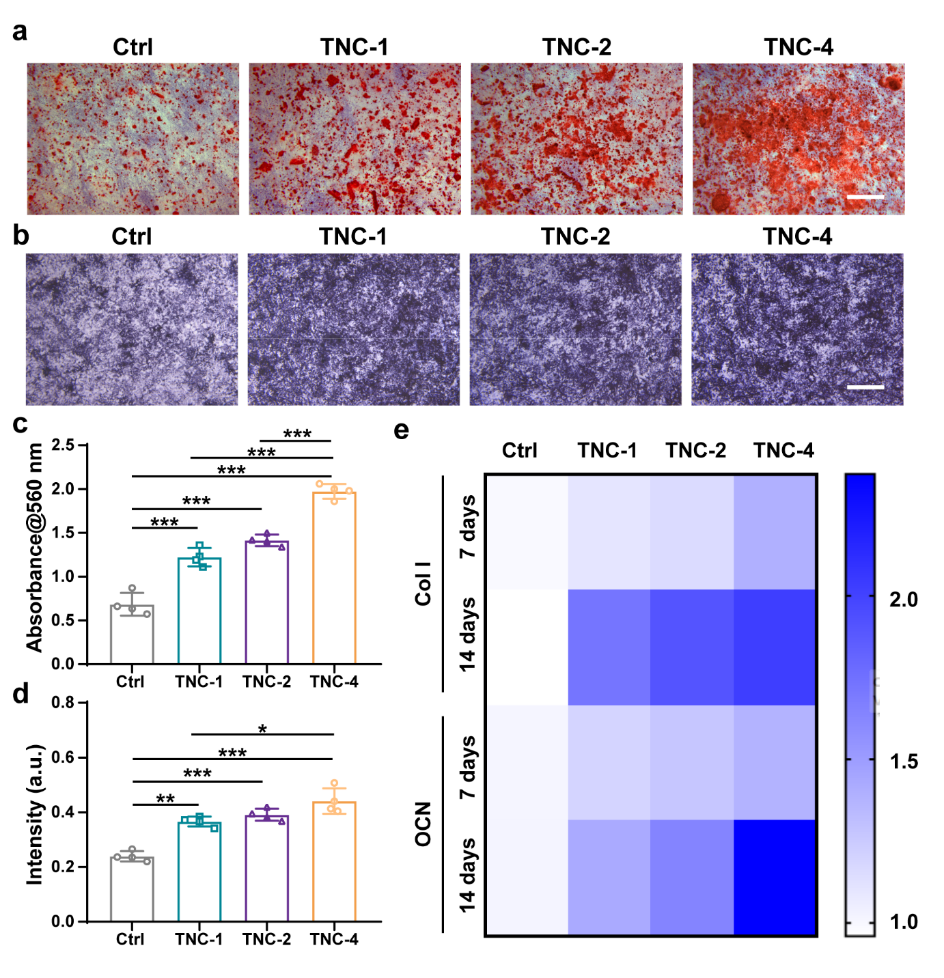


**Figure S15.** Osteogenic inductive property of TNC. (a, c) The representative image (a) and quantitative analysis (c) of alizarin red staining after osteogenic induction for 21 days (n=4), scale bar: 1 mm. (b, d) The representative image (b) and quantitative analysis (d) of alkaline phosphatase staining (n=4), scale bar: 2 mm. (e) The expression of Collagen I (Col I) and osteocalcin (OCN) after osteogenic induction for 7 days and 14 days (n=4). *: *p*<0.05; **: *p*<0.01; ***: *p*<0.001


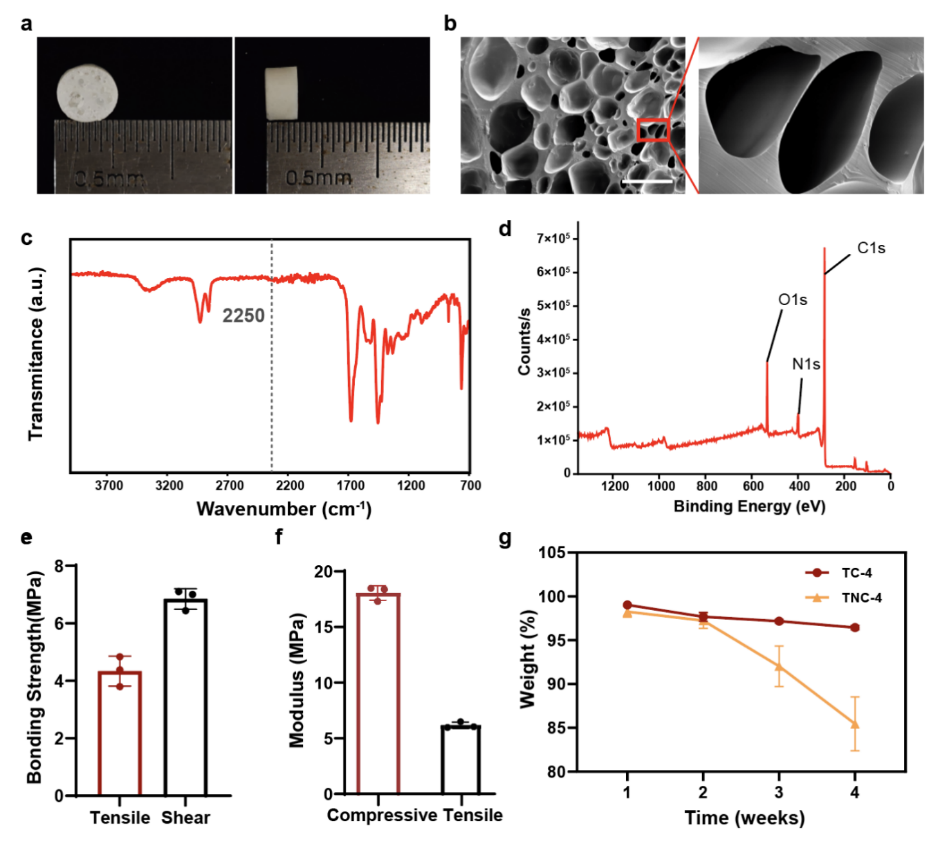


**Figure S16.** The characteristic of TC-4. (a) The representative photograph and (b) SEM image of TC-4, scale bar: 250 μm. The area in red square was further manifested. (c) The FTIR spectra and (d) XPS spectrum of TC-4. (e) The bonding strength and (f) mechanical properties of TC-4. (g) The degradation curve of TC-4 and TNC-4.


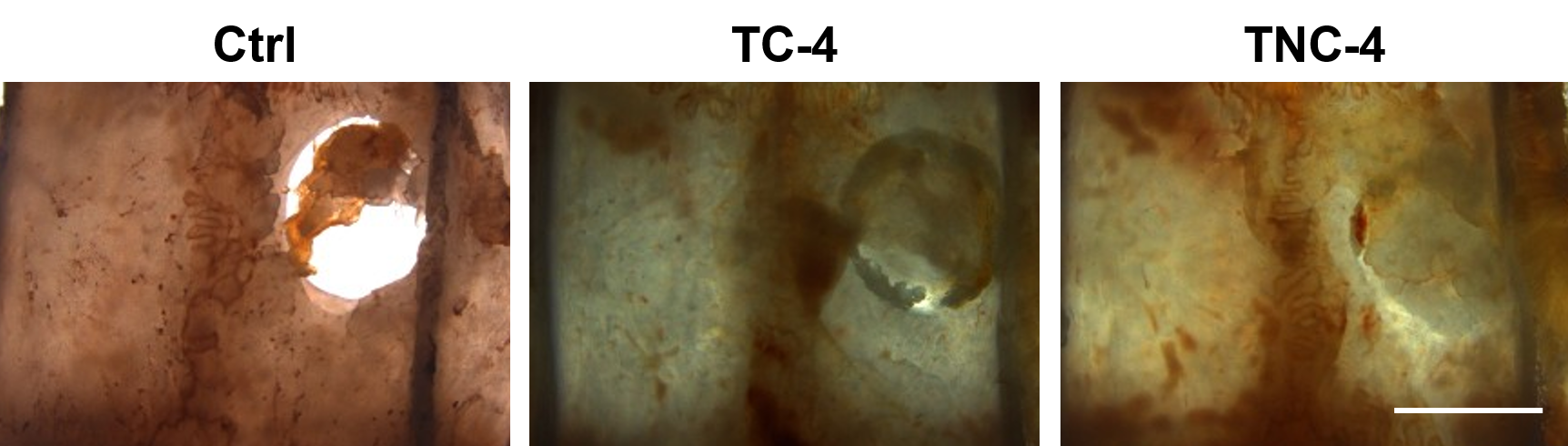


**Figure S17.** The photographs of the skull healed for 8 weeks, scale bar: 4 mm.


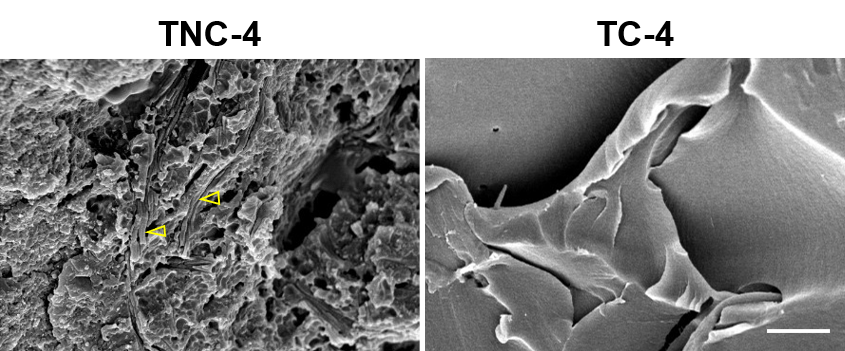


**Figure** **S18**. Representative SEM images of TNC-4 and TC-4 degraded in cathepsin K for 4 weeks. Yellow arrow: exposed collagen. Scale bar: 2 μm.


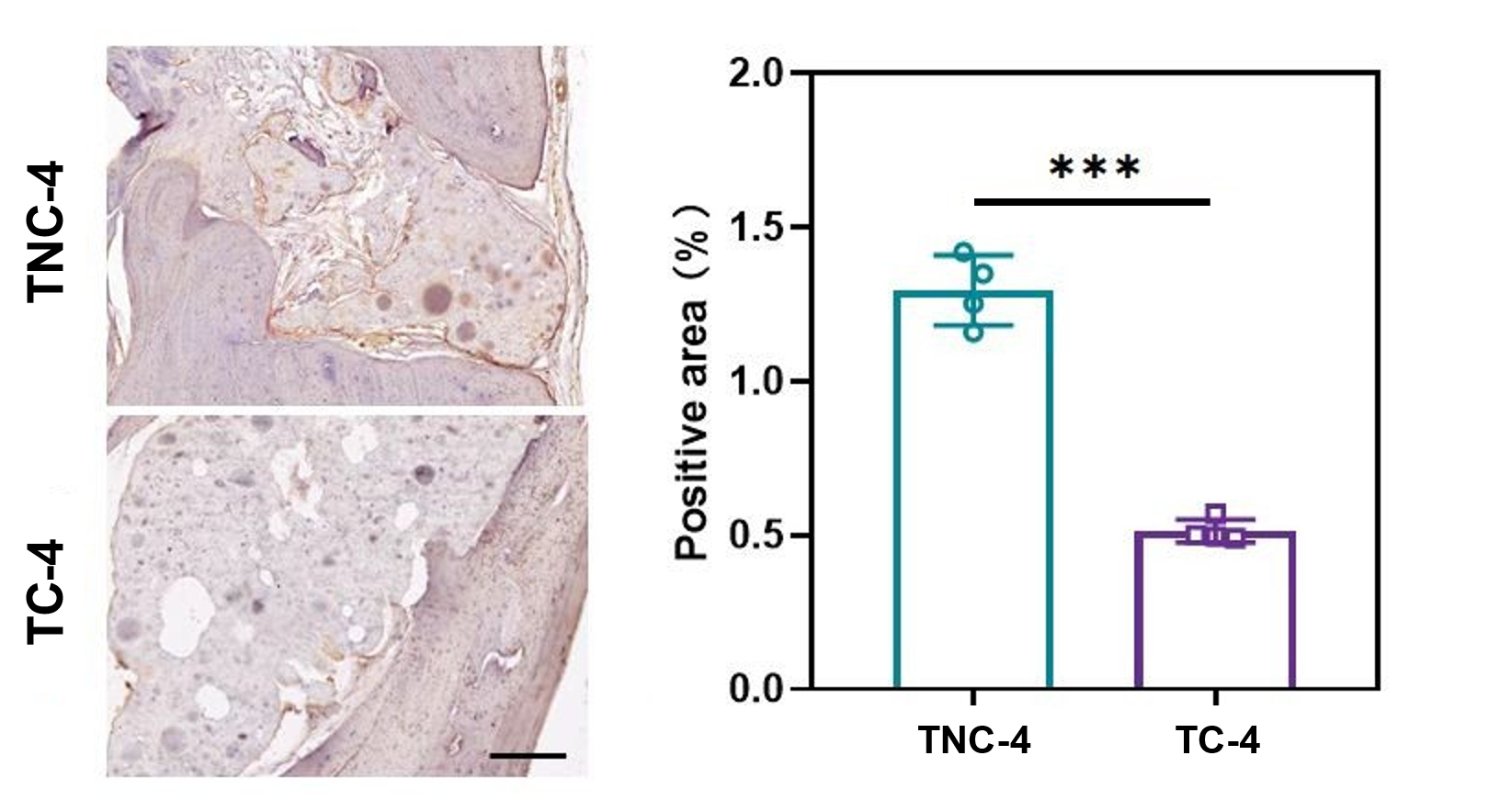


**Figure S19.** Representative TRAP-staining images and quantitative analysis (n=4) after fracture healed for 4 weeks. Scale bar: 100 μm. ***: *p*<0.001.


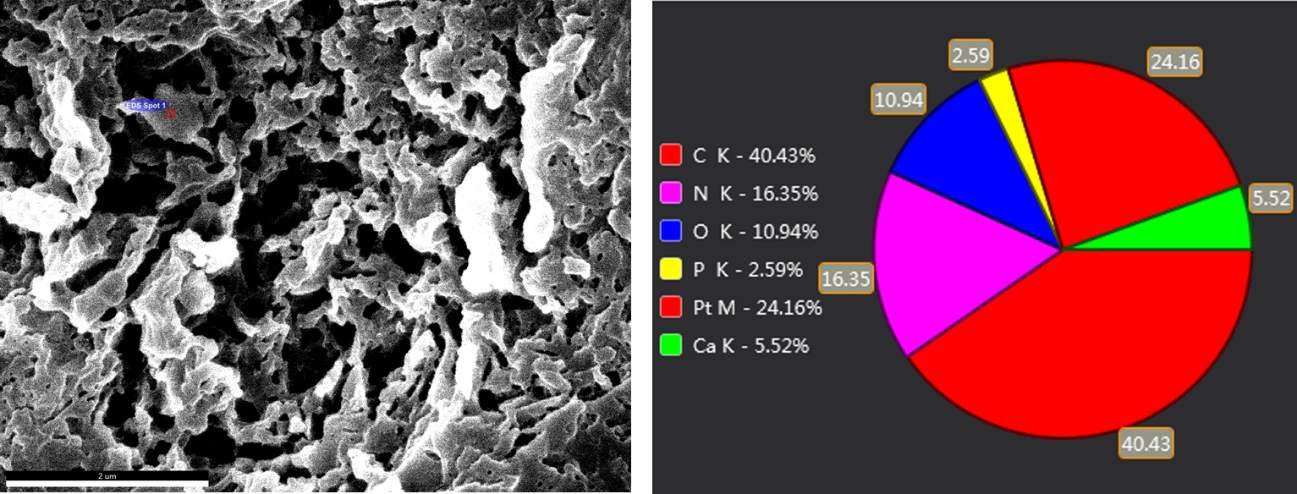


**Figure S20.** SEM-EDX result of mineral layer formed on TNC-4 after 4-week degradation *in vivo*.

**
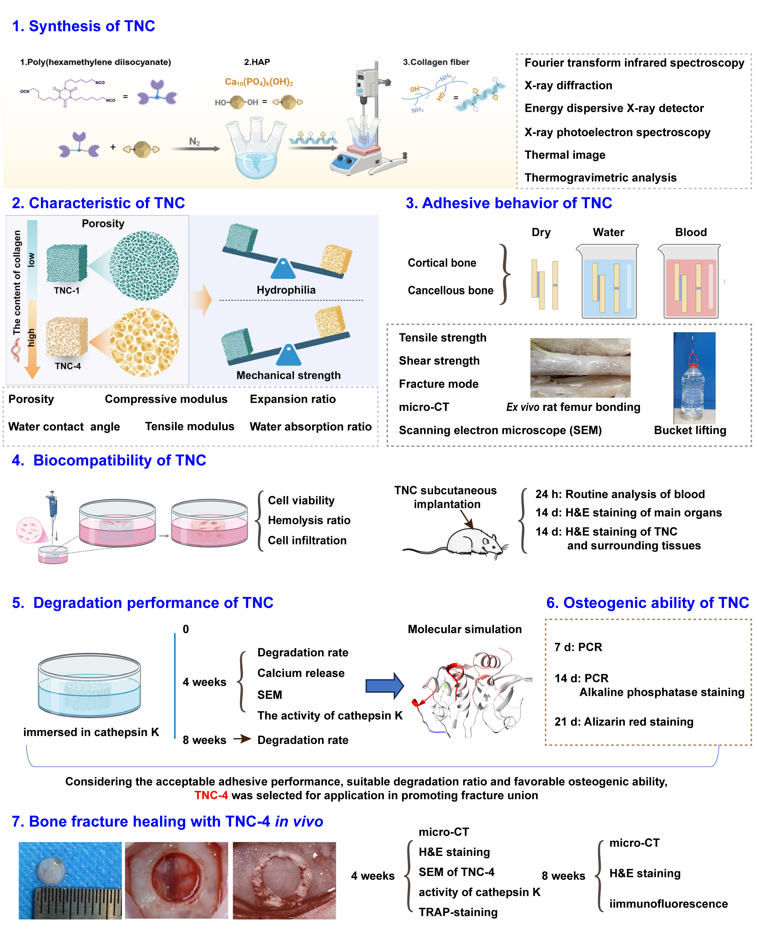
Figure S21.** Flow chart depicting the sequence of experiments conducted in the present study.

**Table S1.** Primer sequences used for RT-PCR.

| Gene | Primer sequence 5’-3’ |
| --- | --- |
| *OCN* | \| F:5’-TGACAAAGCCTTCATGTCCAA-3’ \| \| --- \| \| R:5’-CTCCAAGTCCATTGTTGAGGTAG-3’ \| |
| *Col I* | \| F:5’- AGACCTGTGTGTTCCCTACT -3’ \| \| --- \| \| R:5’- GAATCCATCGGTCATGCTCTC -3’ \| |
| *GAPDH* | \| F:5’-CTGGAGAAACCTGCCAAGTATG-3’ \| \| --- \| \| R:5’-GGTGGAAGAATGGGAGTTGCT-3’ \| |

Abbreviations: Forward, F; Reverse, R

**References：**

1. Hu, S. et al. A Mechanically Reinforced Super Bone Glue Makes a Leap in Hard Tissue Strong Adhesion and Augmented Bone Regeneration. *Adv Sci*. **10**, 2206450 (2023).

2. Li, Q. et al. Band‐Aid‐Like Self‐Fixed Barrier Membranes Enable Superior Bone Augmentation. *Adv Sci*. **10**, e2206981 (2023).

3. Yang, R. et al. Degradable Nanohydroxyapatite-Reinforced Superglue for Rapid Bone Fixation and Promoted Osteogenesis. *ACS Nano*. **18**, 8517–8530 (2024).

4. Choi, S. et al. Bone-Adhesive Anisotropic Tough Hydrogel Mimicking Tendon Enthesis. *Adv Mater*. **35**, 2206207 (2023).

5. Tang, J. et al. Flexible Osteogenic Glue as an All-In-One Solution to Assist Fracture Fixation and Healing. *Adv Funct Mater*. **31**, 2102465 (2021).

6. Yang, R. et al. Tunable Backbone-Degradable Robust Tissue Adhesives Via in Situ Radical Ring-Opening Polymerization. *Nat Commun*. **14**, 1–13 (2023).
